# Resilient math inspired EDA optimized fuzzy adaptive exponent controller for LFC improvement of an EV integrated microgrid

**DOI:** 10.1038/s41598-025-12275-1

**Published:** 2025-08-05

**Authors:** Prakash Chandra Sahu, Buddhadeva Sahoo, Sarat Chandra Swain, Ghanshyam G. Tejani, David Bassir

**Affiliations:** 1 School of Electrical & Computer Science, Indian Institute of Technology, Bhubaneswar, 752050 India; 2 Department of Electrical & Electronics Engineering, SR University, Warangal, 506371 Telangana India; 3 School of Electrical Engineering, KIIT Deemed to be University, 751024 Bhubaneswar, India; 4 Department of Research Analytics, Saveetha Institute of Medical and Technical Sciences, Saveetha Dental College and Hospitals, Saveetha University, Chennai, 600077 India; 5 Department of Industrial Engineering and Management, Yuan Ze University, Taoyuan, 320315 Taiwan; 6 Smart Structural Health Monitoring and Control Laboratory, DGUT-CNAM, Dongguan University of Technology, Dongguan, China; 7 Centre Borelli, ENS -Paris-Saclay University, UMR CNRS 9010, Gif-sur-Yvette, 91190 France

**Keywords:** Islanded microgrid, Electric vehicle, Renewable energy, Math inspired-exponential distribution algorithm, Fuzzy PI-D^Æ^ controller, Storage device, Energy science and technology, Engineering

## Abstract

This study aims to stabilize the frequency of an electric vehicle-integrated AC microgrid in different electrical uncertainties. The recommended microgrid is built by incorporating different distributed generation (DG) oriented power plants. The DG system includes a wind power plant, solar PV plant, diesel generator, fuel cell and geothermal plant. The microgrid frequency goes on oscillating under the action of few uncertainties like dynamics in applied load, fluctuation in wind power and variability in solar power intensity. Further, the charging of electric vehicles extremely disturbs the grid frequency and causes frequency instability issues in the microgrid. This proposed study has anticipated a Fuzzy adaptive exponent PID (Fuzzy PI-D^Æ^) controller to obtain stability in microgrid frequency under different disturbances. Further, the microgrid is associated with different energy-storing devices for improving overall power quality of the system. The Fuzzy PI-D^Æ^ parameters are selected in optimum by incorporating an advanced Math inspired-Exponential distribution algorithm (Mi-EDA) in different operations. The potential of the optimal Fuzzy PI-D^Æ^ controller is compared with fractional ordered fuzzy PID (FO-FPID), Fuzzy PID and PID controllers in concern to the microgrid’s frequency stabilization. The research findings conclude that, the anticipated Fuzzy PI-D^Æ^ approach promptly advances the settling time of frequency by 72.72% and 136.32% and 345.46% to that of FO-FPID, Fuzzy PID and PID controllers respectively. The optimal property of the recommended Mi-EDA technique is compared with the typical sine cosine algorithm (SCA), GA and PSO techniques for validating the potential of the technique.

**Supplementary Information:**

The online version contains supplementary material available at 10.1038/s41598-025-12275-1.

## Introduction

The system for delivering electrical energy from a power plant to an end consumer is the electrical power grid. The numerous generating stations are the origin of the vast power system, while the consumer stands at the end position of the system. The three main energy generation sources for the power system are atomic energy (nuclear power plant), water energy (hydro turbine), and coal energy (steam turbine), in addition to a range of renewable energies. The different forms of renewable energy sources (RES) are in erratic nature and are unable to provide a constant flow of electricity all day. It also contributes to the undesired stability and negative impacts on the whole power system network. The addition of RES causes the system to show high instability as well as low inertia. The network frequency and tie-line power transformation should be maintained nominal to guarantee stable performance. Maintaining equilibrium between net demand and gross electricity generation may make this feasible. A mechanism known as automated generation control (AGC) will be necessary for the power grid in this regard. The AGC targets to govern the total energy production based on load demand^1^. Moreover, it diminishes an error called as the area control error (ACE). The ACE is a joint variation in frequency and tie-line power of the interconnected grid. The error minimization guarantees power system stability in terms of frequency and interline power. The AGC strategy for both single and multi-grid electrical systems has been covered in great detail in a number of academic publications and periodicals^2^. Numerous tiny electrical zones, referred to as control areas, typically make up an interconnected power system. The control zones are situated at variety of places and are divided by interties. To maintain the frequency and active power flow of the system, the AGC monitors the mechanical power of the synchronous generator. Numerous system parameters, including variations in grid frequency (∆F) and tie line power (∆Ptie), must be monitored in order to restore stability. The sum of the many error replies in a given location is the total error, which is easily muffled^3^. The main objective of the integrated grid is to provide reliability and stability in network functional parameters (frequency/tie power) in the event of adverse conditions. Selecting a suitable controller that can provide consistent system functioning and lower ACE can help achieve this^4^. With its own control loop, the AGC action is simply referred to as the principal control loop^5^. The AGC model needs to include more controllers to increase system stability for large signal stabilities^6^. The controller is made more sophisticated by the system connected to renewable energy sources.

The usage of renewable energy sources to produce electricity is growing in the majority of power industries. When numerous green energy-based power plants are electrified at the same time, a microgrid is a local grid^7^. The microgrid is also referred to as a distributed generation (DG) connected network because it is located right to the consumer points^8^. Because of its inertia-less characteristic, the related power producing units are unable to immediately meet rising demand under conditions of extremely high load dynamics. As a result, the microgrid cannot detect stable performance in such load abnormal conditions^9^. Microgrids often use hydrogen gas (fuel cell) power plants, solar electricity, wind energy, and high-speed gas turbines (micro-turbines)^10^. Therefore, fluctuations in solar power intensity and wind speed unpredictability are serious obstacles to microgrid power generation. These environmental dynamic energy-resources (wind/solar), especially when frequency is taken into account, lead to microgrid instability. Microgrids are also connected to a range of energy-storing devices, including flywheels, batteries, and ultra-capacitors, for the prudent supervision of the electricity^11^. The microgrid is functioned in an isolated or grid-integrated fashion to provide power to the end users^12^. The microgrid is able to provide power to the several customers, when it is in stand-alone mode^13– 14^. It is unable to recover from the robust utility grid in an emergency. Extreme grid instability occurs when the system is islanded due to a variety of uncertainties, including load diversions and changes in the strength of wind and solar power^15^. Specifically, the system frequency varies, which makes it difficult to obtain the nominal value in these uncommon situations. However, in grid-connected mode, the microgrid can partially maintain the desired frequency despite of the unpredictable uncertainties and load variability^16^.

**Fig. 1:**
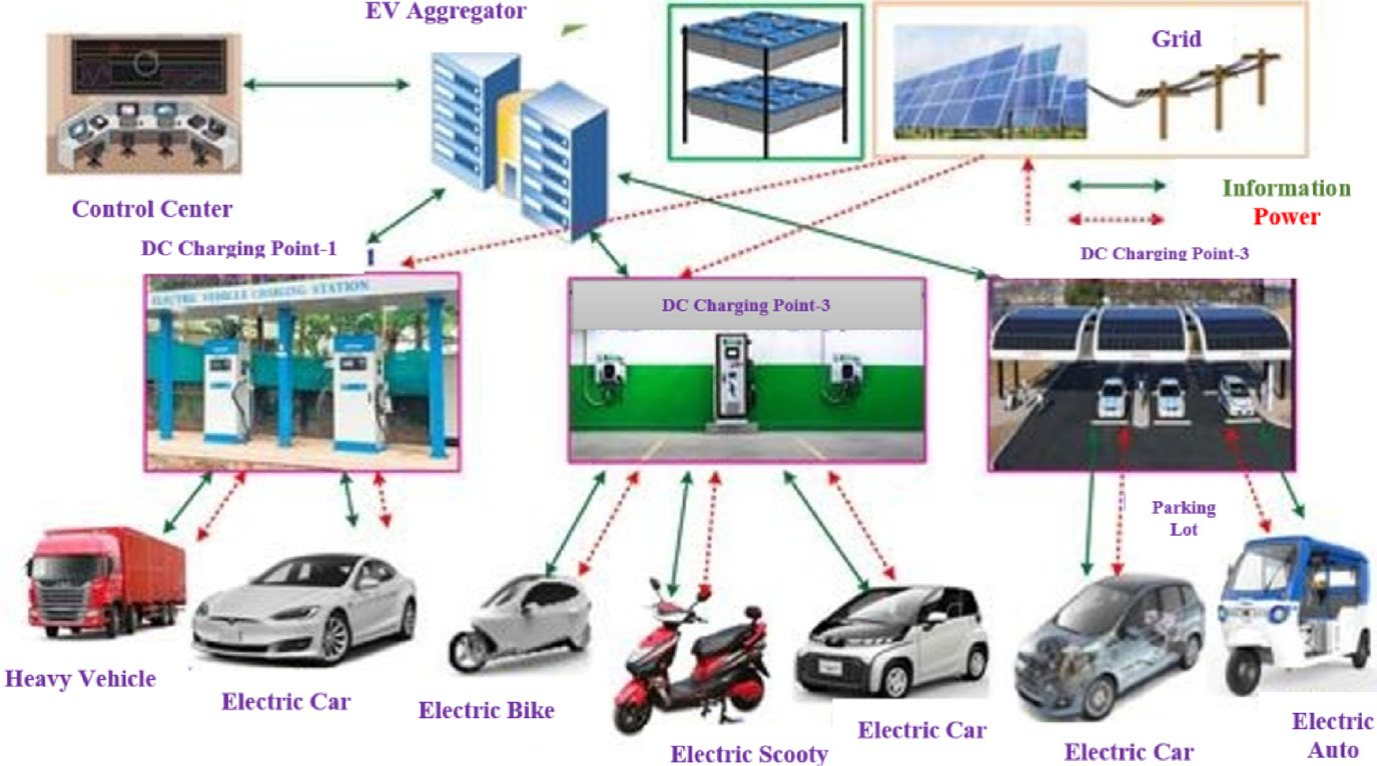
Electric vehicle embedded AC microgrid.

Currently, electric vehicles (EVs) are becoming more and more popular in various transportation sectors due to advancements in the battery industry and their growth^17^. Although microgrids are the only dependable source for inexpensive charging for large numbers of electric vehicles, but the appearance of EVs creates complexity in the system. The basic EV charging station based microgrid structure is visualized in Fig. 1. Furthermore, stability can be improved by utilizing the Electric Vehicle (EV) in its Vehicle to Grid (V2G) mode of power transmission operation^18^. Nonetheless, EVs require a network of charging stations. Given that microgrids are distribution systems, they could provide EV charging stations with a more reliable source of electricity^19^. The charging station based on efficient IGBTs cause harmonics to be felt by the microgrid system. Furthermore, the action of fluctuating demand across the many charging locations causes the grid to become unstable. Adaptive control measures must be taken to suppress undesired disturbances in the system in order to keep the microgrid dynamic and stable amid uncertainty^20^. The carbonless nature of many renewable energy sources, particularly solar and wind power, inspires the creation of a microgrid to power multiple electrical loads^21^. Moreover, the development of electric vehicles motivates the creation of a microgrid model for dependable electric vehicle charging centers. These innovative ideas inspire to model an AC microgrid in inclusion of electric vehicles. This topic selection again encourages the design of several optimal controllers to achieve frequency stability in the system, since the integration of electric vehicles with microgrids causes problems with frequency oscillation.

To achieve frequency stabilization in a microgrid, the controller’s activity and choice of controller is crucial. Different linear controller designs, like wise P, PI, PD, and PID, are utilized to govern the network frequency under a number of dynamic conditions^22^. The simple PI, PID and fractional order PID linear control strategies are completely inferior to develop improved control actions in non-linearity based AC microgrid^23^. As a result, fuzzy logic control, a nonlinear control technique, has been combined with PID and PI control actions to improve stability of the system^24– 25^. Progressively, the fuzzy-based PID controllers are experiencing numerous problems as a result of the microgrid’s heightened uncertainty. Due to the increased degree of uncertainty associated with microgrids, classical fuzzy controllers were unable to perform better in a variety of electrical hazards and adverse weather climates^26^. Consequently, an enhanced fuzzy logic controller called type-2 fuzzy logic control (T2FLC) has been involved with various control actions^27^. Kalyan et al. recommended an intelligent type-2 fuzzy approach for developing advanced LFC function of multi-grid electrical network^28^. The three-dimensional membership structure of this type-2 fuzzy controller allows it to display a broad variety of control actions^29^. Despite having better control over non-linearity in the system, type-2 fuzzy controllers exhibit only moderate control over high uncertainty problems^30^. Inspired by the adaptive exponential of derivative gain and fuzzy ruled control action^31^, , the overall technique encourages to build a novel controller called Fuzzy adaptive exponential PID (Fuzzy PI-D^Æ^) by combining the best aspects of both controllers. The Fuzzy PI-D^Æ^ is a hybrid controller that is expected to stabilize the system’s frequency gracefully of an electric vehicle-injected microgrid. Further, the incorporation of virtual inertia (VI) with the inertia less microgrid gracefully advances the grid stability in several electrical issues^32– 33^.

While the aforementioned controller’s gain variables are defined ideally, they will only expose more effectiveness and their performances will increase abruptly. To enhance the controller’s dynamic behavior, a range of bio-motivated optimization methods are developed recently^34^. Bhatta et al. incorporated an intelligent type-2 graded fuzzy approach for LFC of an electrical system by using a new hybridization based random and harmony search technique^35^. A discrete water cycle algorithm has been intended for optimal sizing of a fuzzy advanced type-II controller in constraint to generation scheduling of LFC based hybrid electrical network^36^. Keeping an eye on LFC expedition, Mishra et al. projected a superior GWO approach for handling advanced optimal process in an AGC system^37^. The optimization strategies outlined above only produce good results when designing controllers with a small number of parameters but needs a large amount of compilation time. This research assignment intended a resilient Fuzzy PI-D^Æ^ controller to build advanced control scenarios in the microgrid model. The controller is elected for its wide controllability and quick error mitigating capability. Further, an improved Math inspired-Exponential distribution algorithm (Mi-EDA) is anticipated for resulting optimal gains of the Fuzzy PI- D^Æ^ controller. The most optimal gain scheduling capabilities and rapid compilation speed make the Mi-EDA approach the preferred choice. Lastly, an extensive tutorial on stability of an electric vehicle integrated microgrid system in connection with several distributed generators (DG) is presented in this research activity. The limitations, advantages and contribution of several published works addressed in this literature review are briefly summarized in Table 1.

**Table 1:** Comparative survey on published works.

Citation No.	Authors Contribution	Innovative/Novelty	Limitations
3 [2021]	Frequency control of a stand-alone microgrid is synthesized with implementing PSO tuned PID controller.	Simplicity in the design of PID controller.	The PID controller fails to provide faster frequency stability under several electrical disturbances. No EV penetration.
5 [2022]	An ideal GTO-governed optimal fractional order PID controller is recommended for AGC of multi-source based electrical grid.	The fractional order PID controller provides advanced stability behavior as compared PID approach	Proposed GTO technique takes long time in the tuning process. FO-PID offers poor performance in non-linear systems.
7 [2022]	An adaptive PSO-governed MPC controller is anticipated for frequency regulation of microgrid.	Model predictive controller offers improved control action in the microgrid frequency control.	The compilation process of the PSO is very time-consuming. PSO offers poor convergence characteristic. No EV penetration
13 [2024]	A multi-stage 1PD-3DOF-PID controller is anticipated for improving LFC of an urban microgrid.	The appearance of the integral pain in the second stage gracefully reduces the steady state error.	The robust study of the 1PD-3DOF-PID controller is missing. No discussion over energy model of the EV.
20 [2023]	LFC function of an AC microgrid is synthesized with incorporating AROA tuned PID Controller.	The compilation time of the proposed AROA technique is very short.	Conventional PID controller takes huge time to stabilize the microgrid frequency. No EV integration.
21 [2020]	An optimal Fuzzy-PID control strategy is recommended for monitoring microgrid frequency.	Recommended Fuzzy-PI controller offers improved control action over PID and FO-PID approaches.	Fuzzy-PI fails to damp out the oscillation due to the absence of the derivative gain. The energy model is missing in PV modeling.
23 [2023]	A self-tuned fuzzy PID controller is suggested for the frequency exercise of an islanded microgrid.	The recommended Fuzzy-PID controller offers faster system stability under step variability in electrical demand.	The study lacks with controller’s resilient study. No EV penetration.

### Contribution of the proposed research study

Overall, the following are the exclusive contributions of this research assignment.

1. The approximated model of the electric vehicle (EV) and the suggested AC microgrid are modelled through transfer function expression.
2. This research study has anticipated a resilient Fuzzy PI- D^Æ^ controller to obtain an AGC control loop in the microgrid system. A metaheuristic Mi-EDA technique is predicted to short out the most conspicuous and fit characteristics of the Fuzzy PI- D^Æ^ controller.
3. The Fuzzy PI- D^Æ^ controller has demonstrated its superiority over FO-FPID, Fuzzy-PID and PID controllers in a number of comparative investigations. Additionally, the suggested Mi-EDA technique demonstrates its superiority over conventional SCA, PSO, and GA approaches in obtaining the controllers’ ideal settings.
4. Multiple sensitivity investigations with broad regulation in system parameters are used to investigate the resilience of the Fuzzy PI- D^Æ^ approach.
5. Finally, the system model and the performances of the proposed approaches are validated through a Real-time based Typhoon-HIL test bench.

### Architecture of the manuscript

The extensive model of the recommended microgrid with EV integration is elaborately specified in Sect. 2. The comprehensive modeling of the robust Fuzzy PI- D^Æ^ approach in several steps is compiled in Sect. 3. The Sect. 4 addresses on basics of objective function. With the use of appropriate flow charts, Sect. 5 provides a thorough lesson on the Mi-EDA approach. Section 6 provides a detailed summary of all research findings, while Sect. 7 discusses the EV-integrated AC microgrid’s conclusion.

## Microgrid modelling in inclusion to electric vehicle

**Fig. 2:**
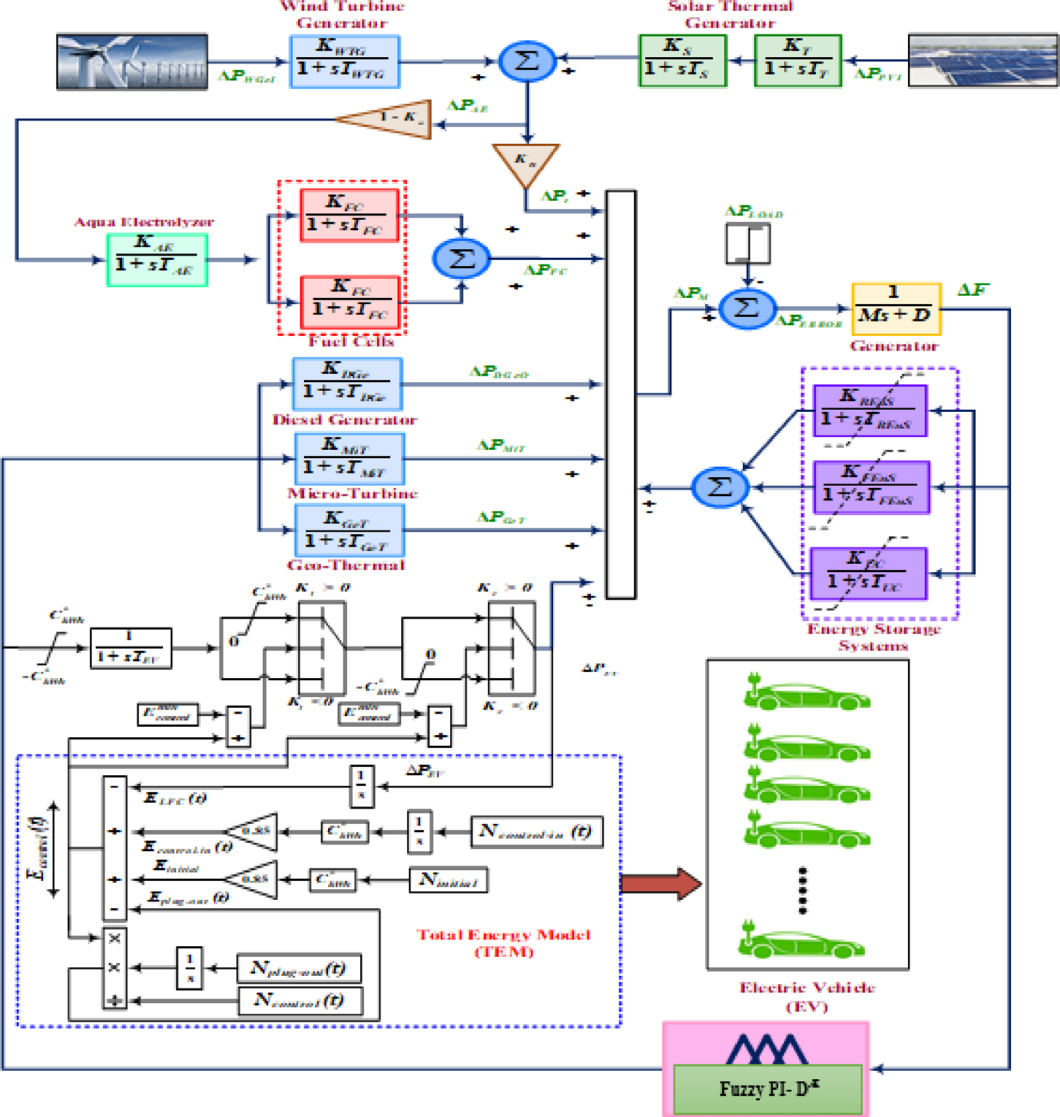
Modelling of electric vehicle penetrated autonomous AC microgrid.

A model that illustrates the modeling of an electrical car in penetration with an AC microgrid is provided in Fig. 2. A distribution grid with many micro sources pierced simultaneously is called a microgrid^38^. The suggested microgrid model for this frequency profile study is essentially a transfer function model that has been organized with the integration of a few electric vehicles, a diesel engine generator (DEG), a micro-turbine (MT), a solar photovoltaic (PV) station, geo-thermal electric station^39^, a wind power platform (WPP), and a fuel cell^40^. To raise the overall rating, the fractional ratings of each generating unit are added to other ratings. The recommended microgrid platform has an average loading of 8.25 MW, with an installed electrical measure of 10 MW. The individual generation capacity of wind-gen. set, solar PV unit, diesel-gen. set, fuel-cell, geo-thermal plant and micro-turbine setup are 2.5 MW, 3.5 MW, 1.5 MW, 1 MW, 1 MW,0.5 MW correspondingly. Frequency oscillation issues arise when the system’s gross demand and total generation are out of balance. Utilizing a few electrical energy-accumulating components, such as flywheels, ultra-capacitors, and battery energy systems, the system’s excess generation is accumulated smoothly.

The following illustrates each transfer function model’s description.

### Wind power station

The doublyfed induction generator and wind turbine work together to convert wind energy into useful electrical energy. Equation 1’s experimental situation suggests that the wind speed is sufficient to generate wind power.

1 $$\:{P}_{W}=\frac{1}{2}.\:C.A.\pi\:.L.S$$

From above, *C* = Characteristic factor, *A* = density of wind in kg/m^3^, *π* = 3.142, *L* = Length of turbine blade in meters, *S* = Wind speed in meters/sec.

An equivalent transfer function expression for the wind generator system is shown in Eq. 2. The transfer function employs single order-based time constant for an approximation model of the wind plant.

2 $$\:{G}_{Wind}\left(s\right)=\frac{\varDelta\:{P}_{WTG}\left(s\right)}{\varDelta\:{P}_{W}\left(s\right)}=\frac{1}{{T}_{WTG}.s+1}$$

In the above expression, *ΔP*_*WTG*_ represents for wind plant output power deviation, *ΔP*_*W*_ stands for input power deviation of the wind plant and *T*_*WTG*_ stands for wind plant time constant.

### PV station

The photovoltaic system generates electricity by utilizing photon rays of solar energy. Unlike wind plant, the solar PV system is also approximated by a single-time constant based first-order transfer function model. The transfer function expression of the PV model is demonstrated in Eq. (3).

3 $$\:{G}_{PV}\left(s\right)=\frac{\varDelta\:{P}_{PV}\left(s\right)}{\varDelta\:{P}_{\varphi\:}\left(s\right)}=\frac{1}{{T}_{PV}.s+1}$$

In this expression, *ΔP*_*PV*_ represents for PV output power deviation, *ΔP*_*ϕ*_ stands for input power deviation of the PV plant and *T*_*PV*_ stands for PV system time constant.

### Diesel generator system

The diesel generator set broadly comprises a governor and turbine section. The governor and turbine sections are approximated with the first-order transfer function model. The transfer function expressions for the governor and turbine are depicted in Eq. (4) and Eq. (5) respectively.

4 $$\:Governor=\frac{\varDelta\:{P}_{V}\left(s\right)}{\varDelta\:{P}_{GO}\left(s\right)}=\frac{1}{{T}_{G}.s+1}$$

5 $$\:Turbine=\frac{\varDelta\:{P}_{M}\left(s\right)}{\varDelta\:{P}_{V}\left(s\right)}=\frac{1}{{T}_{T}.s+1}$$

Here, *ΔP*_*V*_ represents for governor output power deviation or turbine input power deviation, *ΔP*_*GO*_ stands for governor input power deviation and *ΔP*_*M*_ stands for turbine output power deviation. *T*_*G*_ and *T*_*T*_ stands for the time constant of the governor and turbine respectively.

### Micro-turbine system

The Micro-turbine system is a large-speed gas turbine. The model of the micro-turbine is approximated by a single time constant based first-order transfer function model and is depicted in Eq. (6)

6 $$\:{G}_{MT}\left(s\right)=\frac{\varDelta\:{P}_{MTO}\left(s\right)}{\varDelta\:{P}_{MTI}\left(s\right)}=\frac{1}{{T}_{MT}.s+1}$$

In this expression, *ΔP*_*MTO*_ represents for micro-turbine output power deviation, *ΔP*_*MTI*_ stands for input power deviation of the micro-turbine and *T*_*MT*_ stands for micro-turbine time constant.

### Fuel-cell system

The Fuel-Cell is also approximated by single time constant based first order transfer function model and is equated through Eq. (7)

7 $$\:{G}_{Fuel-Cell}\left(s\right)=\frac{\varDelta\:{P}_{FCO}\left(s\right)}{\varDelta\:{P}_{FCI}\left(s\right)}=\frac{1}{{T}_{FC}.s+1}$$

In this expression, *ΔP*_*FCO*_ represents for Fuel-cell output power deviation, *ΔP*_*FCI*_ stands for input power deviation of the fuel-cell and *T*_*FC*_ stands for fuel-cell time constant.

### Electric vehicle designing

The vehicles of the future are thought to be electric vehicles, or EVs. When it comes to emissions-free transportation, clean transportation, etc., it is far superior to traditional vehicles. An approximation design of the EV is viewed as a combination of many battery storage devices in terms of load-end reaction compensation. EVs take part in V2G (vehicle-to-grid) operational actions to speed up frequency stability, depending on the batteries’ state of charge (SOC)^41^. In this instance, SOC with a value 85% is considered to adequate for EVs to participate in the V2G program effectively. EVs will use a lower limit (80% SOC) for their subsequent trip. To extend the life of a storage device, its ideal SOC, which is constantly decreasing during the charging and discharging process, is maintained at 90%. An aggregated model is the most effective method of improving the frequency stability of a multi-source power system because an EV normally affords 10–30 KW. The EV’s aggregate model is displayed in Fig. 2. To provide a synchronized SOC regulator for the EVs, the governor center in this image gets LFC response. In this conceptual, the electric vehicle’s power delivery is remarked by *ΔP*_*EV*_. The power of all governable EVs is represented by the energy signal *Econtrol (t)*, which is also the result of the total energy model (TEM). EVs are eligible to participate in the frequency stability program provided that $\:{E}_{control}^{min}\le\:{E}_{control}\left(t\right)\le\:{E}_{control}^{max}$ The minimum and maximum energies of EVs are represented by $\:{E}_{control}^{min}\:$and $\:{E}_{control}^{max}$ correspondingly. The required quantity of controlled EVs can be computed as follows:

$$\:{N}_{control}\left(t\right)={N}_{initial}+{N}_{control-in}\left(t\right)-{N}_{plig-out}\left(t\right)$$

In this case, $\:{N}_{initial}$ denotes the starting number of EVs, while $\:{N}_{control-in}\left(t\right)$ and $\:{N}_{plug-out}\left(t\right)$ denote the EVs’ respective states upon charging, controllable state, and driving state.

The energy regulated EV’s mean SOC (*SOC*_*avg*_.*)* is computed as

8 $$\:{SOC}_{avg.}=\frac{{E}_{control}\left(t\right)}{{N}_{control}\left(t\right).{C}_{kwh}}\:\times\:100\:\%$$

In the above expression, $\:{E}_{control}\left(t\right)$ represents for the total energy level of the EVs. $\:{N}_{control}\left(t\right)\:and\:{C}_{kwh}$ stands for regulating EV’s number and battery capacity (in kWh) respectively. The detailed modeling of the SOC concept is well demonstrated in several reputed journals^42^.

### Concepts of area control error

The recommended microgrid of this research assignment is a straightforward single-area system, and the error signal it uses is just a change in frequency (*ΔF*). Thus, the employed controller refers to the frequency error signal (*ΔF*) as input and it functions as the total area control error (*ACE*).

## Demonstration over fuzzy adaptive exponent PID (Fuzzy PI-D^Æ^) controller

### Basic adaptive exponent PID (PI-D^Æ^) controller

To improve the response time and transient behaviour, the derivative term (*D*) of the PID controller is to be added with an adaptive exponent (Æ). It is especially useful for reducing response time and speeding up recovery from system disruptions. However, the *D* is frequently left out or set to a value that is almost zero in* PID* controllers. The reason for this omission is that a dampening effect known as “bandwidth-limited derivative control” may be introduced by an increase in *D*. Instability may result from an excessive increase in this term. An additional concern pertaining to D is the “derivative kick.” This issue occurs when there is a sudden shift in the set point, which makes the *D* significantly larger. As a result, the control signal experiences a significant undesired overshoot. On the other hand, the *D* may improve control loop performance. As a result, it is strongly advised that the *D*’s intrinsic problems be resolved while maintaining its advantages.

#### Bandwidth limited derivative control

When high-frequency components in the system cause excessive noise in a process variable measurement, this is known as “bandwidth-limited derivative control”^43^. The controller’s pure derivative operation increased this noise. This problem occurs because the noise is amplified by the high-frequency gain of the pure derivative operation. By looking at a sine wave representation of the system noise, where (*t*) stands for the noise measurement in the * PID* , this impact may be seen.

9 $$\:n\left(t\right)=Asin\left(\omega\:t\right)$$

Making derivative, the expression will be

10 $$\:u\left(t\right)=A\omega\:cos\left(\omega\:t\right)$$

When the high-frequency noise rises by* ω*, the amplification effect is noticeable. Even though this limiting is straight forward and controllable, in real-world situations, an excessively loud control signal may harm actuators and result in subpar actuator performance.

#### Derivative kick effect

A sudden change in the set point results in the “derivative kick,” which makes the *D* unnecessarily big. As a result, the control signal has an undesired and challenging-to-manage peak. The “derivative kick” effect is depicted in Eq. (11) when the set point in a first-order system abruptly changes.

11 $$\:G\left(s\right)=\frac{k}{1+s.T}{e}^{-Ls}$$

Although the derivative value fluctuates until it reaches zero, the *D*’s kicking behaviour is what drives the controller; in other words, this effect is thought to be required for optimal performance. However, because of its uncontrollably high current nature, this signal can seriously impair the functionality and behaviour of any electronic equipment. The controller needs to have a sturdy design. The control system will become unstable if the derivative control signal *D* > 0. On the other hand, the controller becomes insignificant and has no impact on the system’s response if *D* < 0 is negative or falls. A *PI* controller is frequently used to cancel the *D* in order to avoid instability from the kick effect.

#### Motivation for controller design

Using theories of adaptive control and self-calculation of its exponential in the *D*, a novel controller, the *PI-D *-^Æ^ controller, is proposed as a straightforward yet efficient idea to address the two primary issues of the derivative parameter without appreciably increasing the controller’s complexity.

The simplified expression of the conventional PID controller is demonstrated as:

12 $$\:{C}_{P}\left(s\right)={K}_{P}+\:\frac{{K}_{I}}{s}+{K}_{D}\left(s\right)$$

This conventional controller is modified with introducing an additional exponent called adaptive exponent (Æ) in derivative gain (*D*). Then the modified equation is equated as

13 $$\:{C}_{P}\left(s\right)={K}_{P}+\:\frac{{K}_{I}}{s}+{\left[{K}_{D}\left(s\right)\right]}^{\AE\:}$$

The dynamic relationship between the output signal (*s*) and the set point (*s*) yields Æ.

*c*_*1*_ steers clear of uncertainty. A factor (either 1 or 2) and an upper limit (100 is advised) are represented by *c*_*0*_ and *a*_*0*_, respectively.

14 $$\:\AE\:={a}_{0}\left({c}_{0}-\:\frac{y\left(s\right)}{u\left(s\right)+{c}_{1}}\right)$$

Substituting the value of $\:\AE\:$ in Eq. (13), the modified equation is represented as

15 $$\:{C}_{P}\left(s\right)={K}_{P}+\:\frac{{K}_{I}}{s}+{\left[{K}_{D}\left(s\right)\right]}^{{a}_{0}\left({c}_{0}-\:\frac{y\left(s\right)}{u\left(s\right)+{c}_{1}}\right)\:}$$

When it is necessary to change the gain indirectly from *K*_*D*_() without changing the control system’s initial tuning, an auxiliary gain* kaux* (recommended values of 1 or 2) can be applied and the desired expression is given in Eq. (16).

16 $$\:{C}_{P}\left(s\right)={K}_{P}+\:\frac{{K}_{I}}{s}+{K}_{aux}{\left[{K}_{D}\left(s\right)\right]}^{{a}_{0}\left({c}_{0}-\:\frac{y\left(s\right)}{u\left(s\right)+{c}_{1}}\right)\:}$$

Considering the *Kaux* gain as an assignment of 1, and taking only P, I and D terms the controller may be designed as

17 $$\:{C}_{P}\left(s\right)=P+\:I+{D}^{\AE\:}$$

The above expression for the modified PID controller is commonly represented as *PI-D*^Æ^ controller.

By converting the derivative control signal into an exponential function constrained by a specified proportional value, the *PI-D*^Æ^ controller ensures faster reaction times while averting instability. It exhibits two traits: (1) attenuating the derivative response when the system reaches its reference value or experiences low-error disturbances; the cancellation or attenuation technique is used in several control systems. (2) limiting excessive control signals and maintaining a constant response during abrupt set point changes or disturbances; in this first property, it is observed that the exponential response, which gives the derivative controller the ability to improve response times in the control system.

### Basic fuzzy control strategy

Many researchers employ the most well-known controllers, PI and PID, in their respective fields of application. These controllers, which are used in a variety of plants and systems to demonstrate appropriate control actions, provide simple structure and straightforward installation. When proposed in non-linear based systems, these controller performances are significantly deteriorated. Fuzzy logic concepts and principles are then incorporated into the basic PI and PID controllers. Fuzzy PID controllers are created by combining the fuzzy logic notion with a basic PID controller^44^. The fuzzy controller can work for a wide margin of stability because it operates on a wide range of probabilities, even if it only has one clear value. The Fuzzification, the fuzzy inference engine, and the defuzzification conversion process are the three processes in which the fuzzy controller operates.

#### Fuzzification process

**Fig. 3:**
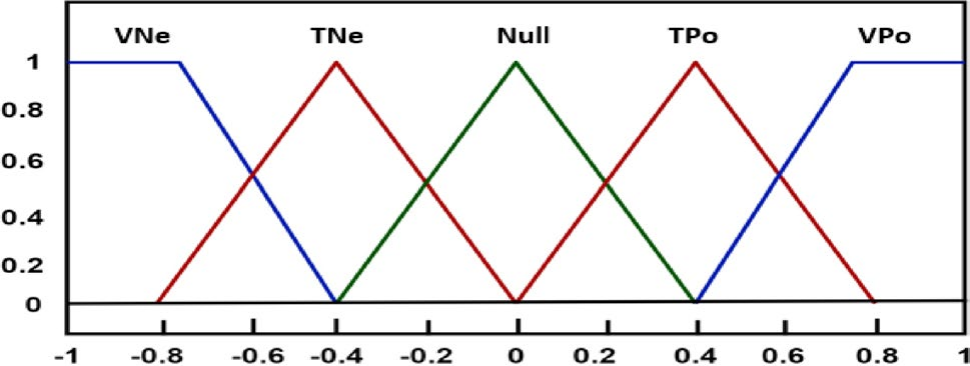
Structure of type-II fuzzy membership function.

Restoring normal system conditions requires a controller to reduce errors. The fuzzy control scheme takes double inputs in order to operate. The error and its derivative are realized as inputs and needed by the fuzzy control actions. This control strategy can only perceive fuzzy standards in order to work. Usually, *ACE* and its derivative are expressed in explicit or in algebraic quantities. The fuzzy control loop method initiates with fuzzification, that assists in converting these unambiguous to the fuzzy formats or fuzzy notations. There are variety membership functions associated with the recommended fuzzy control scheme^45^. The several fuzzy linguistics, such as vast negative (VNe), tiny negative (TNe), Null, tiny positive (TPo) and vast positive (VPo) are assigned as the five different triangular membership functions that are investigated in this study. The membership functions are clearly visualized in Fig. 3.

#### Fuzzy inference engine process

The transitional step of this fuzzy control scheme is named the fuzzy inference engine (FIS). Moreover, the fuzzy variables must be transformed into useful fuzzy sets using if, then based rules^46^. The FIS performs all of these crucial functions for this fuzzy control strategy. The associated rules that have been put into practice for this fuzzy controller are covered in Table 2. This rule states that if *ACE* is vast negative (VNe) and the derivative of *ACE* is tiny negative (TNe), the fuzzy rule’s output will also be vast negative (VNe). Likewise, the five membership function-based fuzzy rules provide 25 results^47^.

**Table 2:** Recommended rules for fuzzy actions.

e^-^	VNe	TNe	Null	TPo	VPo
e					
VNe	VNe	VNe	VNe	TNe	Null
TNe	VNe	TNe	TNe	Null	TPo
Null	TNe	TNe	Null	TPo	TPo
TPo	TNe	Null	TPo	TPo	VPo
VPo	Null	TPo	TPo	VPo	VPo

#### Defuzzification process

Moreover, the fuzzy control strategy needs to give a numerical output response in order for the connected plant to function. The next stage, named defuzzification, translates the fuzzy constraint into the required, distinct (numerical) values using a suitable technique. The defuzzification process of this proposed controller was carried out using the centre of sum (COS) approach.

### Proposed fuzzy adaptive exponent (Fuzzy PI-D^Æ^) controller

**Fig. 4:**
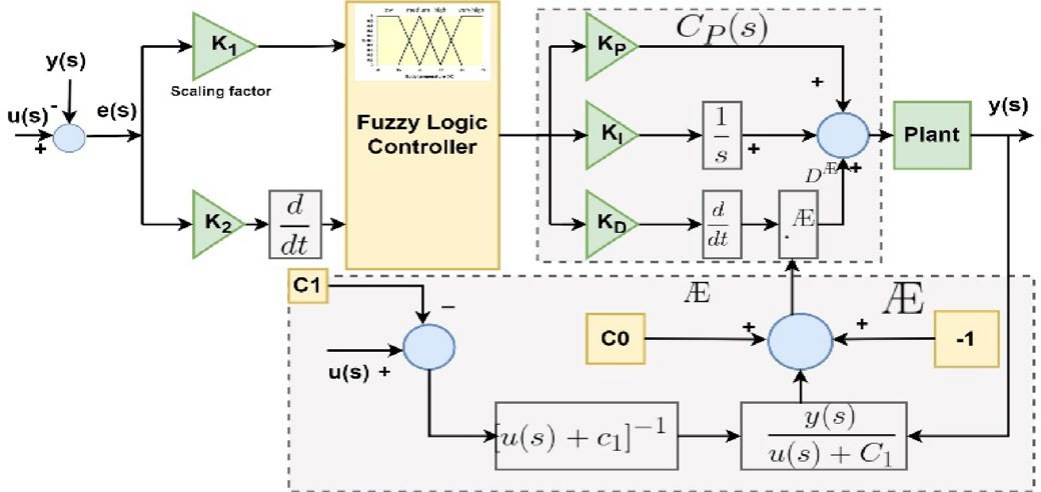
Recommended fuzzy PI-D^***Æ***^ controller’s structure.

The proposed Fuzzy adaptive exponent controller is designed with tracking the features of the ideal adaptive exponent –PID (PI-D^***Æ***^) controller and the conventional fuzzy logic controller. This hybrid Fuzzy PI-D^***Æ***^ controller is able to perform advanced control performances in non-linear electrical systems. The simplified model of the proposed Fuzzy PI-D^***Æ***^ controller is illustrated in Fig. 4.

## Fitness function

Optimization is usually used in conjunction to an appropriate objective tool in engineering applications. The fitness function provides an estimate of the gross error formation over specified time intervals. Selecting the right fitness function is an optimization engineer’s biggest concerns while designing various controllers optimally. Keeping with eye on frequency control problems, the integral of time multiplied absolute error (ITAE), is nominated in this assignment along with the recommended optimization environment. The ITAE can be modeled in one approach as

18 $$ITAE = \int_{0}^{t} {\left( {\sum\limits_{{i = 1}}^{n} {\left| {\Delta f_{i} } \right|} } \right) \cdot t \cdot dt}$$

## Analysis and design of math-inspired exponential distribution algorithm (Mi-EDA)

The math-inspired exponential distribution algorithm (EDA) approach is inspired by an exponential distribution theory. A continuous distribution that is frequently used to describe a range of natural events is the exponential distribution. For instance, the amount of time that will pass before an earthquake occurs possesses an exponential distribution. Furthermore, there is an exponential distribution in time for the likelihood that a car will reach a toll booth. It is common practice to explain past events using an exponential random variable. Formally, it is focused on the amount of time that passes before a particular event takes place^48^. We go over the exponential distribution’s properties and provide its mathematical formulation. Assume that the parameter λ, which may be expressed as x $x\sim A = \pi r^{2}$ Exp(λ), has an exponential random variable x. This random variable’s Probability Density Function (PDF) can be found using

19 $$\:f\left(x\right)=\left\{\begin{array}{c}\lambda\:{e}^{-\lambda\:x\:\:\:\:\:\:\:\:\:\:\:\:\:\:\:\:\:\:\:\:\:\:\:\:\:\:\:\:x\ge\:0}\\\:0\:\:\:\:\:\:\:\:\:\:\:\:\:\:\:Otherwise\end{array}\right.$$

Where, x is the amount of time that passes before an event happens. Since time is continuous, it cannot be negative (x ≥ 0). Furthermore, the exponential distribution’s rate is represented by the parameter λ > 0. The exponential distribution has a parameter λ, as the equation demonstrates. Additionally, the method is used to generate the exponential cumulative distribution function (CDF).

20 $$\:f\left(x\right)=\left\{\begin{array}{c}1-\lambda\:{e}^{-\lambda\:x\:\:\:\:\:\:\:\:\:\:\:\:\:\:\:\:\:\:\:\:\:\:\:\:\:\:\:\:x\ge\:0}\\\:0\:\:\:\:\:\:\:\:\:\:\:\:\:\:\:Otherwise\end{array}\right.$$

The development of the proposed Mi-EDA technique incorporates with different stages. These are demonstrated as follows.

### Initialization stage

We start a population (*Mwinners*) with a collection of *N* randomly generated solutions that have widely distributed values during the startup phase. As a result, we use a group of exponential distributions to explain the search procedure. Every potential solution is treated as an exponential distribution model, and its locations are treated as exponential random variables that follow the model. These solutions are created as vectors of dimension d in the manner described below:

21 $$\:{M}_{winners\_x}=\left[{M}_{winner{s}_{x},1},{M}_{winner{s}_{x},2}\dots\:\dots\:..{M}_{winner{s}_{x},d}\:\right]$$

22 $$\:{M}_{winner{s}_{x},y}\:\in\:(lb,\:ub)$$

$$\:x=1,\:2,\:\dots\:\dots\:.n,\:y=1,\:2,\:\dots\:\dots\:.d$$.

Here, the random variable element *y* of the *x*^*th*^ candidate exponential distribution vector *Mwinners_x* is represented by the notation *Mwinners_x*,* y*. This is how the original population of *Mwinners* can be defined.

23 $$\:{M}_{winners}=\left[\begin{array}{c}{M}_{winners}\begin{array}{c}\:\\\:\text{1,1}\:\end{array},{M}_{winners}\begin{array}{c}\:\\\:\text{1,2}\:\end{array}\dots\:\dots\:\:{M}_{winners}\begin{array}{c}\:\\\:1,d\:\end{array}\:\\\:{M}_{winners}\begin{array}{c}\:\\\:\text{2,1}\:,\:\end{array}{\:M}_{winners}\begin{array}{c}\:\\\:\text{2,2},\:\end{array}{\dots\:\dots\:\:M}_{winners}\begin{array}{c}\:\\\:2,d\end{array}\\\:.\:\:\:\:\:\:\:\:\:\:\:\:\:\:\:\:\:\:\:\:\:\:\:\:\:\:\:\:\:\:\:\:\:\:\:\:\:.\:\:\:\:\:\:\:\:\:\:\:\:\:\:\:\:\:\:\:\:\:\:\:\:\:\:\:\:\:\:\:\:\:\:\:\:\:.\\\:.\:\:\:\:\:\:\:\:\:\:\:\:\:\:\:\:\:\:\:\:\:\:\:\:\:\:\:\:\:\:\:\:\:\:\:\:\:.\:\:\:\:\:\:\:\:\:\:\:\:\:\:\:\:\:\:\:\:\:\:\:\:\:\:\:\:\:\:\:\:\:\:\:\:\:\:.\\\:{M}_{winners}\begin{array}{c}\:\\\:n,1\:,\:\end{array}\:\:\:{M}_{winners}\begin{array}{c}\:\\\:n,1\:,\:\end{array}\dots\:\dots\:\:{M}_{winners}\begin{array}{c}\:\\\:n,d,\:\end{array}\\\:.\\\:.\end{array}\right]$$

Each exponential random variable of the candidate exponential distribution in the issue space can be generated at random using the following equation:

24 $$\:{M}_{winnersx,y}=lb+rand.(ub-lb)$$

In the above expression, the range [*lb*, *ub*] is used to construct each member, *Mwinnersx*,* y*. The problem’s lower and upper bounds are determined by *lb* and *ub*, respectively. A random number generated in the interval [0, 1] is denoted by the symbol *rand*.

### Exploitation stage

The different features of the exponential distribution model, including the memoryless property, exponential rate, standard variance, and mean, are used in the exploitation phase. To direct the search process to the global optimum, a guiding solution is also used. Many algorithms investigate the search space surrounding good solutions by drawing in inferior ones because the area surrounding a good solution holds promise for locating the global optimum^49^. The global optimum around the guiding solution is thus what we look for. The mean of a sorted population’s first three best solutions is the guiding solution (*Mguide*), which is computed as follows:

25 $$\:{Mguide}^{time}=\:\frac{{M}_{winners\_best1{.}^{time}}+{M}_{winners\_best2{.}^{time}}+{M}_{winners\_best3{.}^{time}}}{3}$$

Here, the guiding solution at iteration (time) is determined by *Mguide*^*time*^. The waiting period until the subsequent event takes place is represented by the random variable in the exponential distribution. As a result, going forward, the term “time” is used in place of “iteration”. The exploitative optimization model uses winners and losers to update the existing new solution that deviates from the exponential distribution. We make the assumption that in order to update the new solution ($\:{V}_{x}^{time+1}$),

26 $$\:{V}_{x}^{time+1}=\left\{\begin{array}{c}u.\left({memoryless}_{x}^{time}-{\sigma\:}^{2}\right)+v.{Mguide}^{time}\:if\:{Mwinners}_{x}^{time}={memoryless}_{x}^{time}\\\:v.\left({memoryless}_{x}^{time}-{\sigma\:}^{2}\right)+\text{log}\left(\varnothing\:\right).{Mwinners}_{x}^{time}\:\:\:\:\:\:\:\:\:\:\:\:\:\:\:\:\:\:\:\:\:\:\:\:\:\:\:\:\:\:\:\:\:\:\:\:Otherwise\end{array}\right.$$

27 $$\:u={\left(f\right)}^{10}$$

28 $$\:v={\left(f\right)}^{5}$$

29 $$\:f=2*rand-1\:$$

From the above expression, ‘*Φ*’ is a random number and $\:{memoryless}_{x}^{time}$ is the memoryless matrix’s *x*^*th*^ solution.

uniformly created in [0, 1], with *f* being a random integer generated in [− 1, 1] and *u* and *v* being adaptive parameters.

### Exploration stage

The suggested algorithm’s exploration phase is demonstrated in this subsection. The algorithm’s exploration phase finds the promising areas of the search space that are thought to have the global optimum solution. Two winners from the initial population that follow the exponential distribution are used to construct the optimization model for the EDA exploration phase. The following equations are used to update the new solution.

30 $$\:{V}_{x}^{time+1}=\:{Mwinners}_{x}^{time}-{M}^{time}+(c.{Z}_{1}+\left(1-c\right).{Z}_{2})$$

31 $$\:{M}^{time}=\:\frac{1}{N}.\sum\:_{x=1}^{N}{Mwinners}_{y,x}^{time},\:\:\:\:\:y=1,\:2,\:\dots\:\dots\:d.$$

From the above expression, the mean of every solution found in the initial population is denoted by *M*^*time*^. It is calculated by dividing the population size by the total of all exponential random variables that fall into the same dimension. The adjusted parameter *c*, which is defined as follows, indicates the proportion of information shared between the *Z*_*1*_ and *Z*_*2*_ vectors and the current new solution:

32 $$\:c=d*f$$

33 $$\:d=\frac{1-time}{Max\_time}$$

In this case, *d* is an adaptive parameter that begins at zero and is progressively decreased over time. The current time and the entire course of time are denoted by time and *Max*_time. Promising vectors *Z*_*1*_ and *Z*_*2*_ are produced by:

34 $$\:{Z}_{1}=M-{Q}_{1}+{Q}_{2}$$

35 $$\:{Z}_{2}=M-{Q}_{2}+{Q}_{1}$$

$$\:{Q}_{1}=\:M-{Mwinners}_{rand.1}$$

36 $$\:{Q}_{2}=\:M-{Mwinners}_{rand.2}$$

**Fig. 5:**
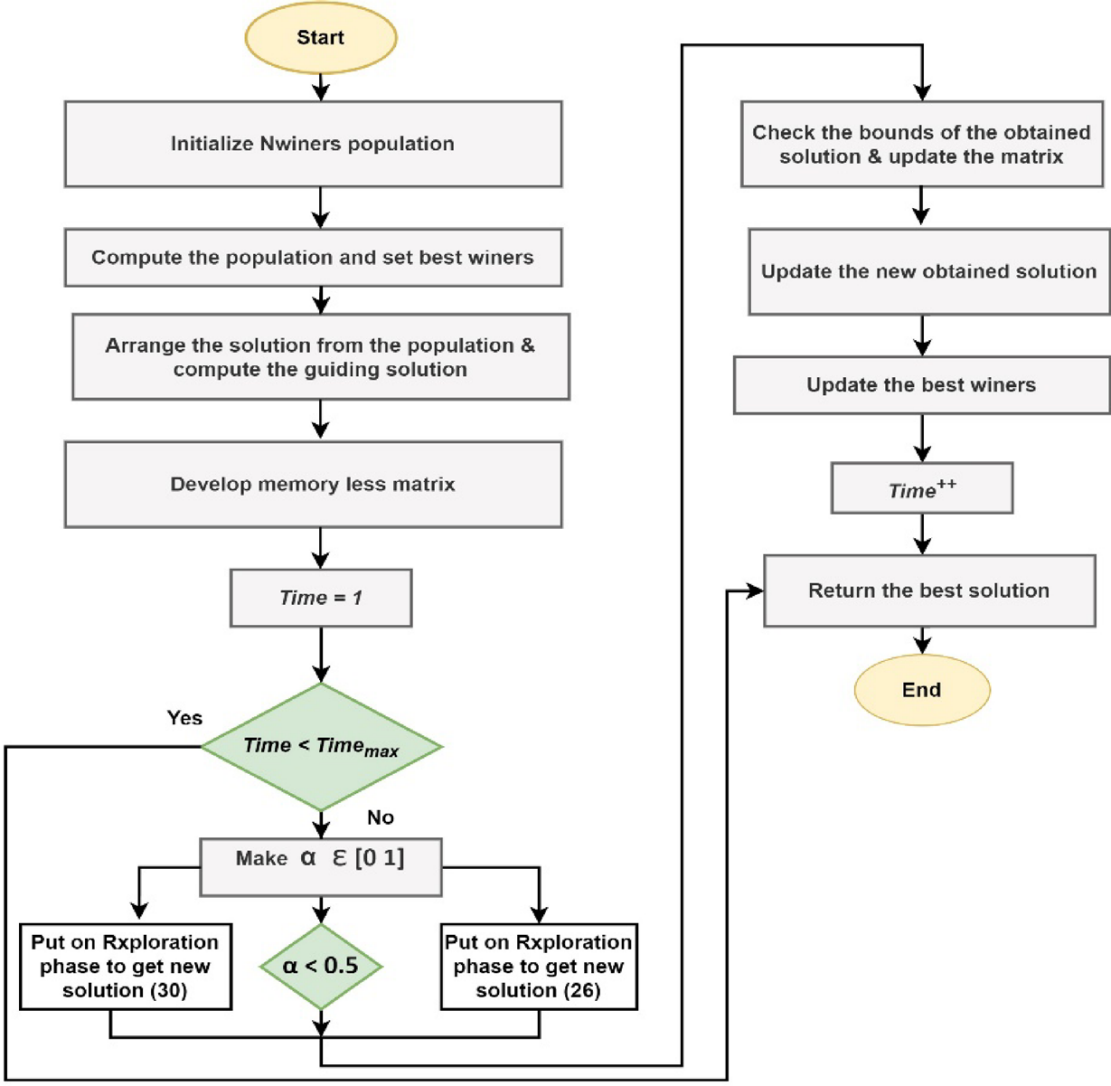
Math-inspired exponential distribution algorithm (Mi-EDA).

Additionally, *Q*_*1*_ and *Q*_*2*_ indicate the distance between the mean solution and *Mwinners.rand*_*1*_ and *Mwinners.rand*_*2*_, which are winners in terms of the exponential distribution chosen at random from the initial population^50^. The developed flow chart for this proposed EDA technique is illustrated in Fig. 5.

## Discussion over results and findings

This section presents the usefulness of the recommended Mi-EDA based Fuzzy PI-D^Æ^ technique for the purpose of frequency stabilization of an autonomous AC microgrid in inclusion to electric vehicles. The assignment on the investigation of frequency stability has been put together under a variety of disturbances, including uncertainty in solar power, wind speed variations and load fluctuations. The predicted Mi-EDA algorithm and performance validation of the suggested Fuzzy PI-D^Æ^ controller support the uniqueness of this research endeavor. In the first, the potential of the proposed Fuzzy PI-D^Æ^ controller is compared to the performances of several existing controllers, including the PID, Fuzzy PID, and FO-FPID controllers. In the next, the optimization behavior of the suggested Mi-EDA method is compared to a few typical algorithms, including the PSO, SCA, and GA algorithms. The MATLAB platform is used to develop the single-area microgrid model using a PC with an i-7 processor and 8GB of RAM. The codes for the anticipated Mi-EDA technique are written in the.m file and are executed simultaneously with the microgrid model. The model then uses the computationally customized gains of the control loops to extract different performance outcomes of the smart grid. Lastly, some findings and conclusions demonstrate the effectiveness of the proposed Fuzzy PI-D^Æ^ controller and the originality of the Mi-EDA technique.

### Controller’s efficacy analysis

It is vigorous to explore the capabilities of the recommended Fuzzy PI-D^Æ^ approach for resuming the frequency monitoring of the EV integrated single area stand-alone microgrid. For this dealing, the suggested Fuzzy PI-D^Æ^ controller’s performance has been combined with that of the FO-FPID controller, Fuzzy PID controller and the conventional PID control schemes in coordination with the frequency governing. The Table 3 shows the Mi-EDA governed optimal parameters of the anticipated Fuzzy PI-D^Æ^ and basic Fuzzy PID controllers in five different executions. A 2% step load perturbation is used during the optimization procedure. The PID controller and the FO-FPID controller are also optimized in the Mi-EDA optimization scenario for comparative study. In the end, the microgrid model implements each controller’s optimal gain independently to produce a variety of system dynamic reactions. The intelligent tool named ITAE is incorporated to the optimization method to obtain the most acceptable gains of the recommended control scheme.

**Table 3:** Mi-EDA based tuned parameters of fuzzy PI-D^Æ^ controller in five different runs.

Runs/ Algorithms/ Approaches	Mi-EDA optimized Fuzzy PI-D^Æ^ Controller K_1_ K_2_ K_*P*_ K_I_ K_D_ ITAE	Mi-EDA optimized Fuzzy PID Controller K_1_ K_2_ K_*P*_ K_I_ K_D_ ITAE
First run	0.108 0.886 1.684 0.868 -0.808 0.1442	0.772 0.228 -1.086 0.556 -0.788 2.382
Second run	0.224 0.298 1.928 1.568 -0.905 0.1608	0.906 0.792 0.778 -1.021 -1.326 2.022
Third run	0.892 0.708 1.998 1.390 -1.889 0.1546	0.028 0.326 -0.675 -1.043 -1.004 2.884
Fourth run	0.780 0.228 0.906 0.894 -0.898 0.1806	0.878 0.802 1.768 0.822 -0.902 2.290
Fifth run	0.206 0.118 1.002 1.606 -1.004 0.1382	0.492 0.118 1.002 1.006 1.562 2.998

**Fig. 6:**
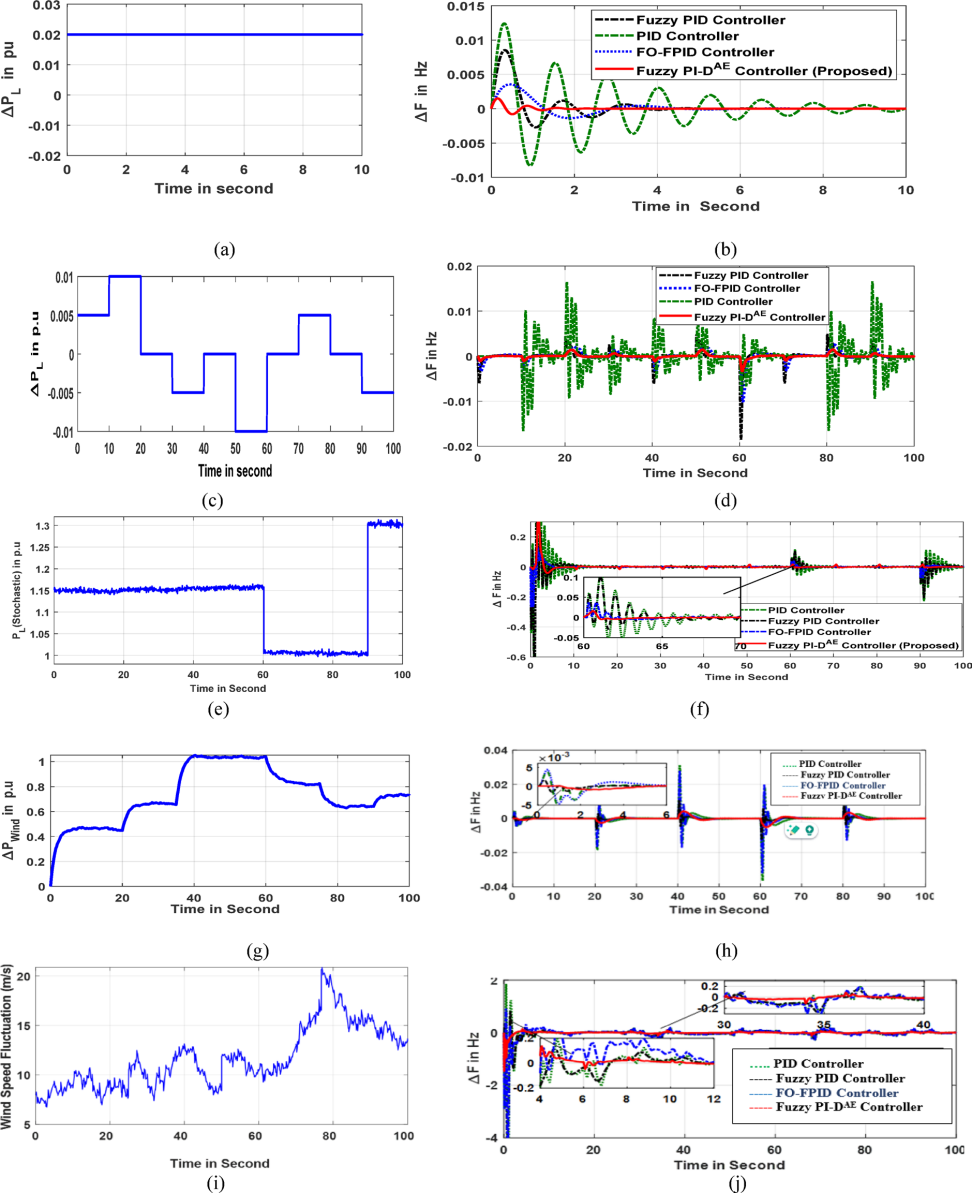
(**a**) Step response of 2% (**b**) Microgrid frequency response (**c**) Random load (**d**) Frequency deviation due to random load (**e**) Stochastic load (**f**) Frequency disturbance due to stochastic load (**g**) wind fluctuation (**h**) Frequency oscillation response (**i**) Realistic wind fluctuation (**j**) Frequency response under realistic wind fluctuation.

The microgrid model implements each of the aforementioned controllers separately to create system dynamic responses, particularly for variations in frequency and power generation. The stability study is synthesized under a step load disturbance of 2% as shown in Fig. 6a. The resulted error responses are framed together for making transparency in the validation study. In concern to this, the Mi-EDA optimized Fuzzy PI-D^Æ^, FO-FPID, Fuzzy PID and PID simulated dynamic response of microgrid frequency is shown in Fig. 6b. In addition, the viability of the controllers are examined under random and stochastic load fluctuations as presented in Fig. 6c and Fig. 6e respectively. Under random load dynamics, the error response of the microgrid frequency is depicted in Fig. 6d, however the response of dynamics in microgrid frequency under stochastic load disturbance is given in Fig. 6f. The response of a time-variant wind fluctuation uncertainty is given in Fig. 6g. Under this wind uncertainty, the extracted response of microgrid frequency is depicted in Fig. 6h. The different performance parameters such as time of settling (St), overshoot (O. St) and undershoot (U. St) of microgrid frequency response are compared analytically in a tabular manner as given in Table 4. The graphical and comparative analysis of the outcomes justifies the excellent behavior of the Fuzzy PI-D^Æ^ controller for gaining rapid stability in microgrid frequency. The anticipated Fuzzy PI-D^Æ^ approach has proved to be the most important candidate for maintaining stability in frequency. It is critical noticed from the frequency diversion response (ΔF1) that the projected Fuzzy PI-DÆ controller promptly reduced the settling time of ΔF1 b by 72.72% and 136.32% and 345.46% to that of FO-FPID, Fuzzy PID and PID controllers respectively.

**Table 4:** Efficacy measures of the response *∆F*.

Controllers			Fuzzy PI-D^Æ^			Fuzzy FO-PID			Fuzzy PID		
Sl. No	Disturbance	Responses	O.St×10^**− 2**^ ( in p.u.)	U.St×10^**− 2**^ ( in p.u.)	St (in sec.)	O.S t×10^**− 2**^ ( in p.u.)	U.St×10^**− 2**^ ( in p.u.)	St (in sec.)	O.St×10^**− 2**^ ( in p.u.)	U.St×10^**− 2**^ ( in p.u.)	St (in sec.)
11	Load	*∆F*	0.202	-0.102	2.528	0.462	-0.214	4.204	0.828	-0.342	5.226
12	R.load	*∆F*	0.226	-0.412	3.212	0.424	-0.560	4.606	0.526	-0.588	5.878
13	S.load	*∆F*	28.852	-4.772	5.894	18.692	-24.548	6.402	22.904	-42.114	8.556

Further, the effectiveness of the proposed microgrid model and efficacy of the anticipated Fuzzy PI-D^Æ^ approach are investigated under the action a realistic based wind speed fluctuation as given in Fig. 6i. The response of monitoring in grid frequency profile in this realistic wind speed fluctuation is visualized in Fig. 6j. Critically examining on the deviated frequency response validates for erudite behavior of this Fuzzy PI-D^Æ^ scheme in coordination with frequency profile improvement of microgrid. The minimal oscillation and the faster settling time are key factors for obtaining advanced frequency stability.

### Superiority justification of Mi-EDA approach

**Fig. 7:**
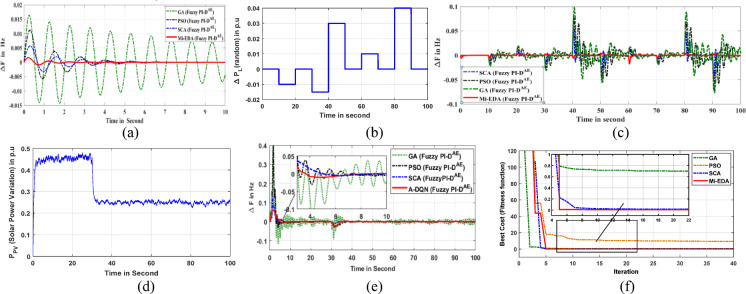
(**a**) Microgrid frequency change under step load (**b**) Random load (**c**) Frequency deviation due to random load (**d**) Solar power fluctuation (**e**) Frequency deviation under solar power disturbance (f) Convergence curve.

The performance and the resiliency attributes of the recommended controllers are mostly determined by the optimization algorithm. A novel Mi-EDA algorithm is recommended for this frequency regulation assignment to choose the Fuzzy PI-D^Æ^ controller’s parameters. Frequency dynamic responses are used to compare the Mi-EDA algorithm’s performance to that of the original SCA, PSO, and GA approaches to conclude it’s preeminence. Figure 7a displays the variation in grid frequency oscillation by Mi-EDA, SCA, PSO, and GA in a typical Fuzzy PI-D^Æ^ control action under step load disturbance. Additionally, the random load and solar power fluctuation disturbances are used to validate the optimization techniques, which are then displayed in Figs. 7b, d respectively. Figures 7c, e show, respectively, the deviating image of the grid frequency under random load dynamics and solar power uncertainty. The intelligent outcomes make it conclude that the advised Mi-EDA monitored Fuzzy PI-D^Æ^ controller produces system stability faster than SCA, GA, and conventional PSO algorithms.

Once more, a convergence curve has been used to analyze the Mi-EDA algorithm’s performance, and the results are shown in Fig. 7f. The curve illustrates how the Mi-EDA technique exhibits faster convergence behavior when compared to alternative optimization algorithms, resulting in the common ITAE scenarios. It is experimentally verified that, proposed Mi-EDA approach gracefully advances the fitness function ITAE value by 70.74% and 227.18% as compared to SCA and PSO approaches respectively.

### Effectiveness of G2V & V2G operation on microgrid operation

**Fig. 8:**
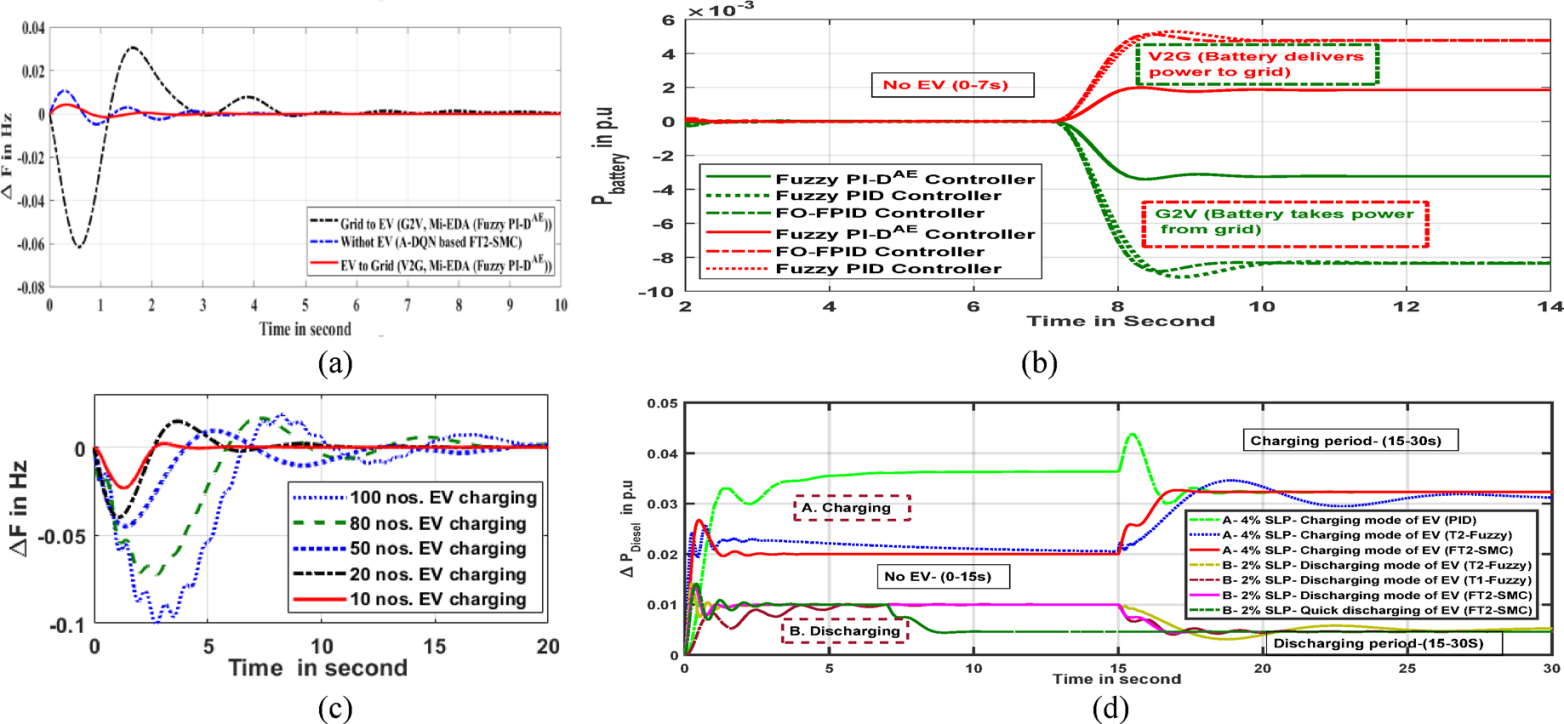
(**a**) Oscillation in grid frequency in different actions of EV (**b**) Dynamic response of EV battery output power in various conditions (**c**) Oscillation in grid frequency under group EV charging (**d**) Per unit power oscillation of diesel generator.

The penetration of intelligent EVs in AC microgrids has a considerable influence on the performance of all DGs in the microgrid, especially when they are charging. The EV functions in two different ways when it is integrated with the base microgrid. These operational functions are V2G (vehicle-to-grid) for battery discharging and grid-to-vehicle (G2V) for battery recharging. Electric vehicles (EVs) can use power electronics converters to enable electricity to respective batteries by referring to microgrids as the electrical resource. Usually, EV seeks for charging while it demands electricity for its battery after a lengthy run or consistently drives. In this operational scenario, the EV simply operates as a load and pulls electrical energy from the microgrid to electrify the battery to its SOC band. This circumstance of EV integration substantially impacts grid frequency and starts diverging from its rated value. As a result, the controllers step in immediately to prevent frequency oscillation and attempt to swiftly return to a constant and rated frequency. However, in the situation of overloading or dynamic load situations, a fully charged EV can provide power to the grid to fulfill the deficit. In these situations, the EV helps to improve power quality and can sustain frequency stability when a secondary controller is in place. The Fig. 8a visualizes the microgrid’s frequency performance when operating in three distinct EV modes. Due to EV charging (G2V), the frequency error response oscillates more and is settled by the controlling action of the proposed Fuzzy PI-D^Æ^ controller. This shows the system frequency goes on large deviation under EV charging conditions. The only time the system frequency varies normally is due to the step load uncertainty. At this operation, no EV is applied to it. When compared to the EV charging scenario, the variance is negligible. However, in an EV discharging (V2G) scenario, the frequency deviation is minuscule. When in V2G mode, the EV works with the secondary controller as a source to help eliminate the frequency disturbance gracefully.

The Fig. 8b shows the reaction of the per unit (p.u) EV battery output power under various operating conditions. Although EV is not integrated during the first seven seconds, it comes under electrification (charging/discharging) over the following seven to fourteen seconds (7–14 s). When as V2G action, the installed EV battery starts transporting power to the parental microgrid and reacts as discharging. The symbolic notation in microgrid clarifies the mode of the power transformation. The positive sign is an indication of inward flow of power, however the outward flow of power is notified by the negative symbol. As the number of electric vehicles increases, so does the charging power. Figure 8c visualizes the oscillatory frequency profile of the microgrid under the consideration of the mass of EV charging. The Simulink platform yields all of the dynamic responses, and a novel Mi-EDA based Fuzzy PI-D^Æ^ controller is suggested. The outcome shows that the number of EVs that need to be introduced to the grid is inversely correlated with frequency response stability. The diesel generator’s output power per unit in the context of charging and discharging an electric car is shown in Fig. 8d. During the EV charging phase, the diesel generator needs to supply the grid with more electricity than it does during the discharging phase. The power analysis of the diesel generator is carried out for 2% and 4% step load disturbances, correspondingly. The diesel generator affords energy for step load dynamics through the period of 0–15 s, during which no EV is integrated into the microgrid. The EV comes into action during a time period of 15–30 s in the microgrid scenario. The delivered power from the DGe (diesel generator) starts to adapt to the demands as soon as the EV is penetrated. The proposed fuzzy PI-D^Æ^ controller does a decent job of maintaining stability in the power transfer under these adverse scenarios.

### Expansion of research with incorporating hybrid system

**Fig. 9:**
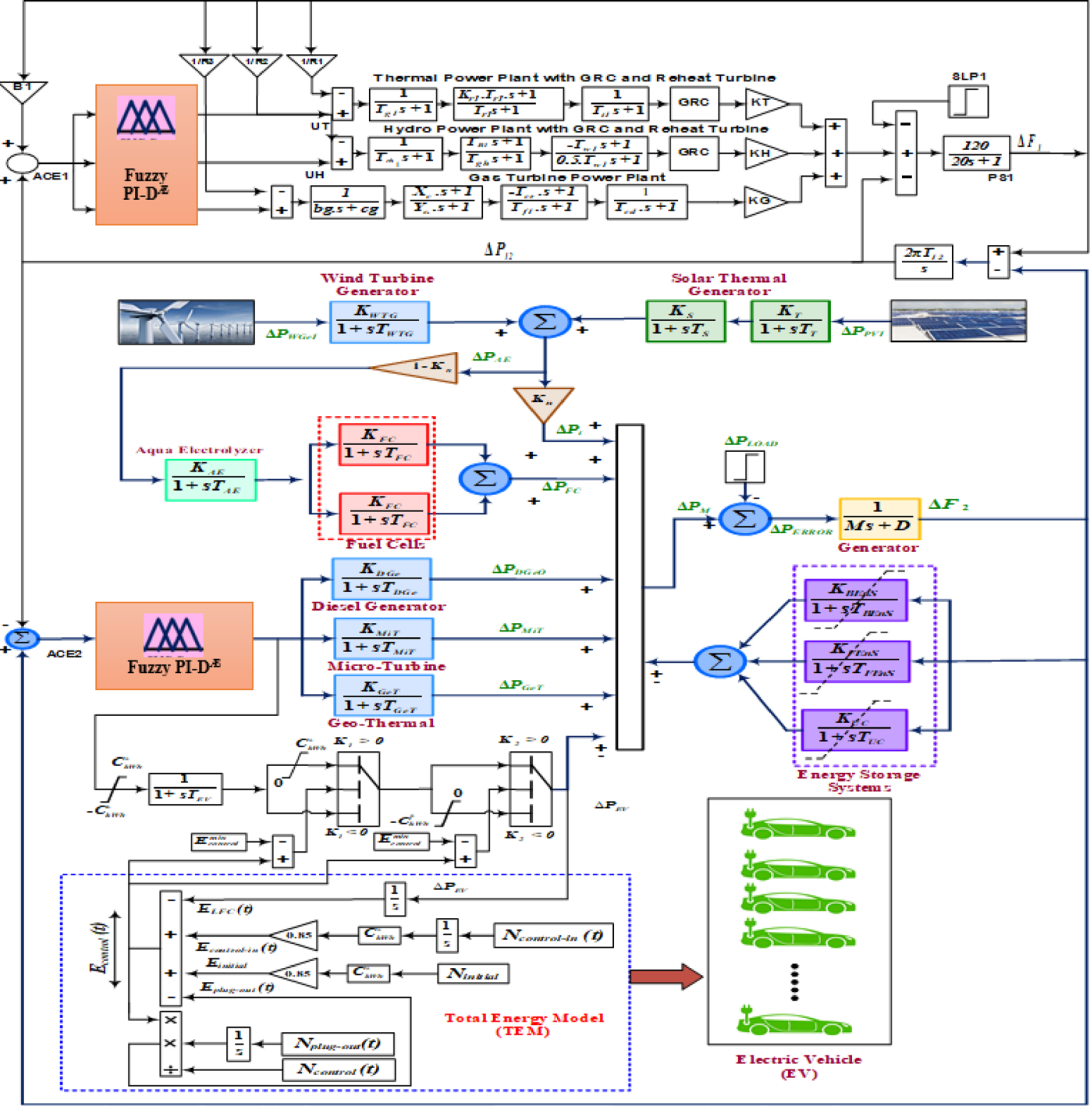
Hybrid power system model in inclusion to electric vehicles.

The research assignment on frequency stability is further expanded to a two-area hybrid-microgrid system. The control action of the hybrid-microgrid system is monitored with approaching robust Fuzzy PI-D^Æ^ controller. The Fig. 9 depicts the comprehensive transfer function model of the proposed hybrid-microgrid system. A thermal station, hydro station, and gas power station are all included in the control area 1 of this proposed hybrid system. Nonetheless, the suggested model’s control region 2 is comprised of many power plants that rely on renewable energy sources and are interconnected with one another. Equational transfer function expressions are used to mathematically design each power-generating unit separately^46^. In order to evaluate stability, the system is modeled with the addition of electric vehicles and various uncertainties. Electric vehicles put a strain on the grid in two distinct operational scenarios. The EV often arrives for charging in order to prepare it for usage whenever needed in the future. Here, the EV just serves as a load, drawing power from the grid. Because the EV charging indicator in the model draws electricity from the grid, for which it is represented by the negative (-) sign. When the EV is completely charged, it will be powerful enough to be used for transportation; otherwise, it can be employed as a generating asset and supply electricity to the grid at times of high demand or emergency. When an EV is in a grid-discharging condition and injects electricity into the grid as needed, it displays a positive (+) sign. The controller parameters are chosen as the best optimum with employing a sophisticated Mi-EDA technique.

*Comprehensive discuss over results and findings*

A Simulink software is used in the research process to create the models of the hybrid microgrid system and anticipated Fuzzy PI-D^Æ^ controller. The .m file of the MATLAB 2022 is used to build the necessary coding of the suggested Mi-EDA method. This research work incorporates a unique optimization strategy and a robust controller, therefore the performances of both approaches have been analyzed under various loading conditions. In order to build the necessary control action, the suggested Fuzzy PI-D^Æ^ controller is also designed in Simulink software and then connected with the microgrid model. Through the use of the bode plot approach, the analysis is advanced in tandem with the discussion of the controller performance study, algorithm performance study, sensitivity study and stability study. Further, a real-time based Typhoon-HIL test bench is utilized to aware frequency stabilization study of the proposed hybrid microgrid for exposing strong validation of the proposed approaches.

#### Controller’s potential validation

In a MATLAB environment, the controllability and robustness of the proposed Fuzzy PI-D^Æ^ controller are examined. The optimal settings of the suggested Mi-EDA tuned Fuzzy PI-D^Æ^ controller are compiled in Table 5, together with fitness function values.

**Table 5:** Mi-EDA tuned scheduled parameters of the fuzzy PI-D^Æ^ Controller.

Technique/ Controller	Units	Mi-EDA based Fuzzy PI-D^Æ^ approach					ITAE
		K_1_	K_2_	K_*P*_	K_I_	K_D_	
Area-1	Thermal	0.1218	0.3926	1.7046	0.9066	-0.7944	4.0218
	Hydro	0.5682	0.8562	1.7980	1.0026	-1.0282	3.1292
	Gas	0.9882	0.4428	0.8042	0.8988	-0.8978	3.8856
Area-2 (Microgrid)		0.4080	0.6002	1.3028	1.8002	-1.9898	3.5844

**Fig. 10:**
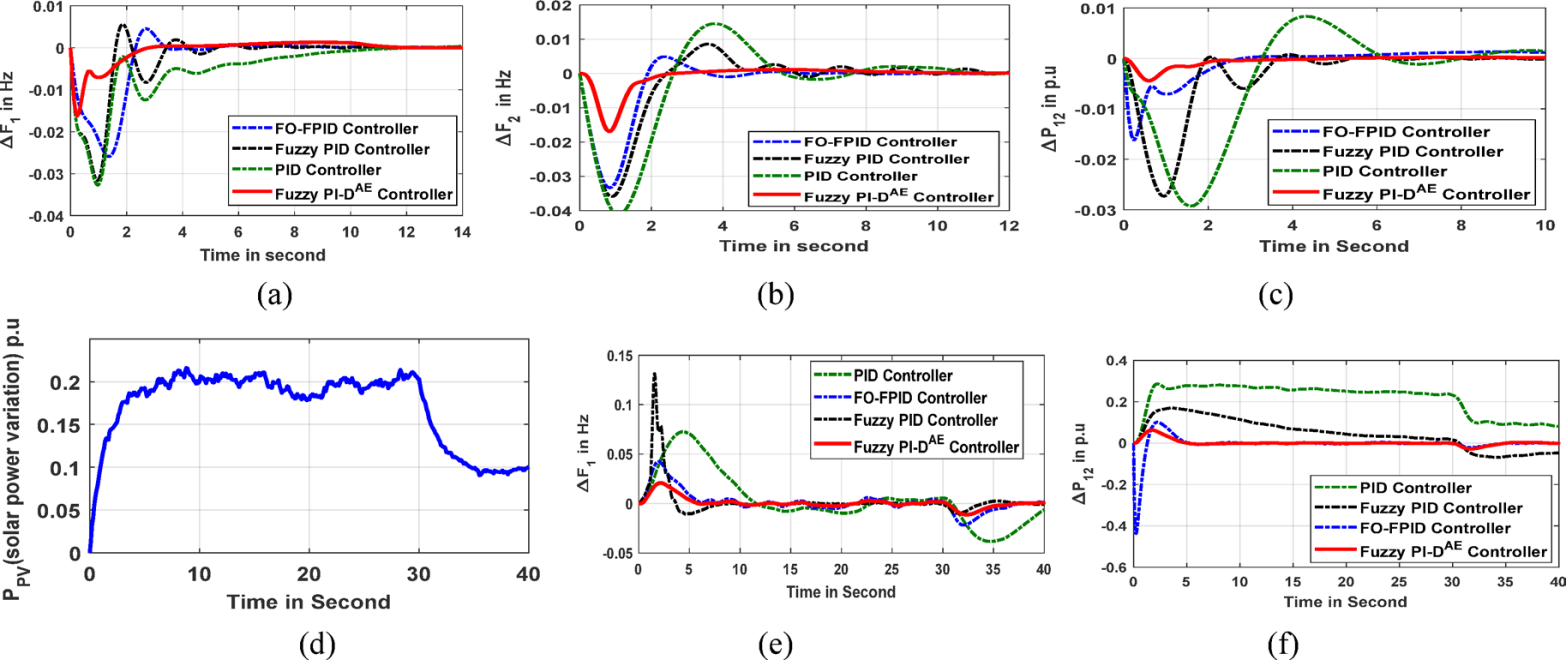
(**a**) Oscillation in area1 frequency (**b**) Oscillation in area2 frequency (**c**) Inter-line power deviation (**d**) Solar power variation (**e**) Oscillation in area1 frequency due to solar power disturbance (**f**) Tie-line power oscillation due to solar noise.

The verification of the suggested Fuzzy PI-D^Æ^ controller’s performance over Fuzzy FO-PID, Fuzzy PID and PID is conducted in several disturbance scenarios. The advanced Mi-EDA algorithm has been used to optimize the parameters of the aforementioned controllers. Each controller’s performance is tracked separately to ensure that the system variables i.e. frequency deviation and tie-line power fluctuation stay within predetermined bounds. In order to provide clarity in the comparative analysis, the time domain simulated responses of the grid by these suggested controllers are developed in different situations. The investigation is initiated with an electrical disturbance of 2% SLP at area1. The Mi-EDA optimized Fuzzy PI-D^Æ^, Fuzzy FO-PID, Fuzzy PID, and PID controller monitored simulated responses of area1 frequency (*ΔF*_*1*_) and area2 frequencies (*ΔF*_*2*_) are illustrated in Fig. 10a and Fig. 10b respectively. The responses are stabilized on the occurrence of a step load dynamics. Figure 10c shows the tie-line power that resulted between area 1 and area 2. The controller’s potential is also testified under a stochastic-based solar power fluctuation as displayed in Fig. 10d. The dynamic simulated responses of area1 frequency error and interline power transfer due to solar power variation are shown in Fig. 10e, f respectively. In conclusion, Table 6 compiles a range of index metrics, including overshoot, undershoot and steady-state time of the interline power and frequency responses. Examining all simulated images and index parameters points to the Fuzzy PI-D^Æ^ approach’s outstanding performance in achieving rapid stability in a variety of system signals while adhering to EV integrated hybrid microgrid system constraints.

**Table 6:** Performance metrics of the microgrid response (Fig. 10).

Controllers		Fuzzy PI-D^Æ^			Fuzzy FO-PID			Fuzzy PID		
Sl.No	Responses	O.St×10^**− 2**^ ( in p.u.)	U.St×10^**− 2**^ ( in p.u.)	St (in sec.))	O.St×10^**− 2**^ ( in p.u.)	U.St×10^**− 2**^ ( in p.u.)	St (in sec.)	O.St×10^**− 2**^ ( in p.u.)	U.St×10^**− 2**^ ( in p.u.)	St (in sec.)
1.	*∆F*_*1*_	0.0110	-1.6276	3.8212	0.4276	-2.2088	6.6412	0.5246	-3.2048	8.2288
2.	*∆F*_*2*_	0.0182	-1.8522	4.6044	0.5454	-3.2912	7.8206	0.7182	-0.1414	8.6026
3.	*∆P*_*12*_	0.0022	-0.4066	2.5682	0.0012	-1.2826	4.6438	0.0028	-2.5922	8.4792

A thorough examination of each outcomes included in Table 6 demonstrates that the recommended Fuzzy PI-D^Æ^ controller is of higher quality for reducing undesired system disturbances. The enhanced performance contracts for a least steady-state time with negligible oscillations. The method that allows for a shorter steady state time is a sign that the grid frequency will quickly recover from any disturbances.

#### Proposed algorithm’s efficacy validation

When it comes to the stability research of the proposed EV-based microgrid power system, the optimal property and dynamic nature of the recommended Mi-EDA algorithm has to be examined over the original SCA, PSO, and GA algorithms. The potential of the Mi-EDA approach is investigated through several dynamic situations.

##### Situation in step load dynamic

**Fig. 11:**
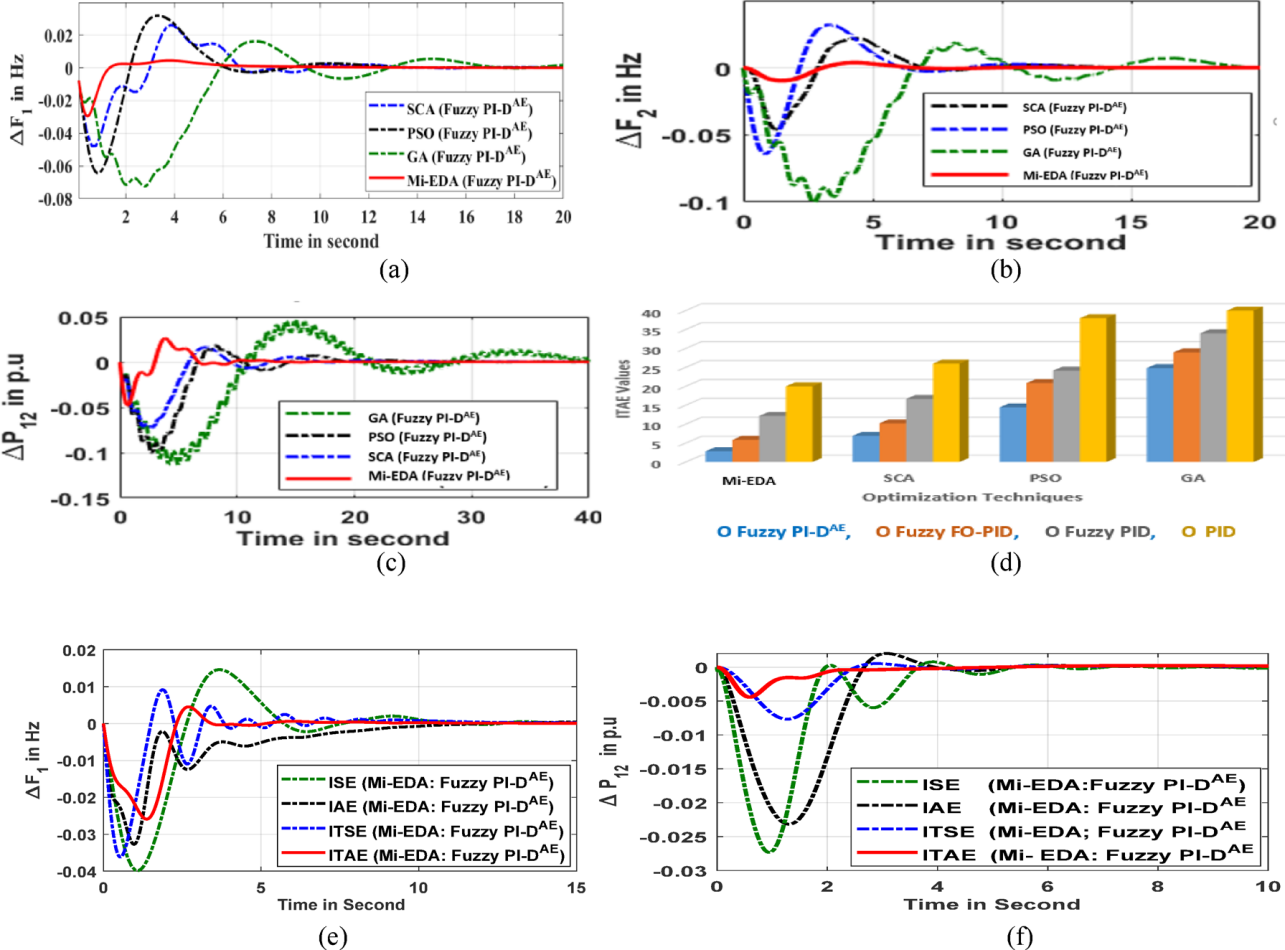
(**a**) Oscillation in area1 frequency (**b**) Oscillation in area2 frequency (**c**) Dynamic response of inter-line power flow (**d**) Graphical view of ITAE values (**e**) Oscillation in area1 frequency (f) Oscillation in tie-line power.

In order to cause disruptions in the system performances, a step load of 2% is applied in region 1 in this validation study. Algorithms like Mi-EDA, SCA, PSO, and GA are used under common Fuzzy PI-D^Æ^ scenario, to find optimal controller settings and subsequently provide steady performance in the system. The reaction of area1 and area2 frequencies in such stabilization operations is shown in Fig. 11a and Fig. 11b respectively. Additionally, Fig. 11c shows the tie-line power stability response.

Table 7 compiles the efficiency indexes such as steady state time, crest undershoot and crest overshoot of above discussed frequency and tie-line power responses. The Fig. 11(d) shows a graphical representation of ITAE values using various techniques. A thorough analysis of stability parameters and dynamic responses supports the Mi-EDA approach’s exceptional performance in determining the ideal Fuzzy PI-D^Æ^ controller parameter for achieving increased system stability.

**Table 7:** Performance metrics of *∆F*_*1*_, *∆F*_*2*_ and *∆P*_*12*_.

Sl. no.	Algorithm	∆F_1_			∆F_2_			∆*P*_12_		
		O.St ×10^− 2^ (in *p*.u.)	U.St×10^− 2^ (in *p*.u.)(-)	St (in sec.)	O.St×10^− 2^ (in *p*.u.)	U.St×10^− 2^ (in *p*.u.)(-)	St (in sec.)	O.St ×10^− 2^ (in *p*.u.)	U.St×10^− 2^ (in *p*.u.)(-)	St (in sec.)
1	*Mi-EDA*	0.1282	2.1164	5.82	0.0086	1.8280	7.28	3.1856	5.4422	10.86
2	*SCA*	2.2468	4.2296	11.64	2.6644	6.1936	12.46	2.5498	6.9032	17.52
3	*PSO*	3.4644	6.4094	14.86	1.8832	4.6288	13.28	2.8016	10.2644	18.60
4	*GA*	1.1056	7.2888	17.48	1.4068	10.5074	18.56	4.8056	14.4692	38.46

Further, the significance of the ITAE objective function over few additional objective functions such as ITSE (integral of time multiplied squared error), IAE (integral of absolute error) and ISE (integral of squared error) is compared to justify its resiliency. In common Mi-EDA: Fuzzy PI-D^Æ^ control scenario, all above-discussed objective functions are employed individually and the corresponding area1 frequency stabilization response is depicted in Fig. 11(e) while the regulated tie-line power deviation response is visualized in Fig. 11(f). It is clearly identified from both responses that recommended ITAE objective function is more effective in amending quick stability in microgrid behavior under a step load disturbance.

#### Typhoon-HIL based Real-time experimental setup

The experimental setup of this research work includes a 32GB RAM based desktop with fully loaded Typhoon-HIL software. The model of the proposed microgrid system as well as suggested Fuzzy PI-D^Æ^ controller is developed in this software. The Typhoon-HIL device is interfaced with the desktop through USB port. The HIL device is opted for the Real-time action while simulating the model in typhoon software platform. A suitable digital storage oscilloscope (DSO) is interpreted with the setup to take different dynamic responses of the system.

**Fig. 12:**
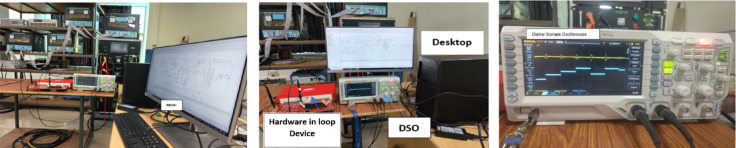
Experimental setup for (**a**) MG model with Fuzzy PI-D^Æ^ controller controller (**b**) Step load frequency study (**c**) Random load study.

**Fig. 13:**
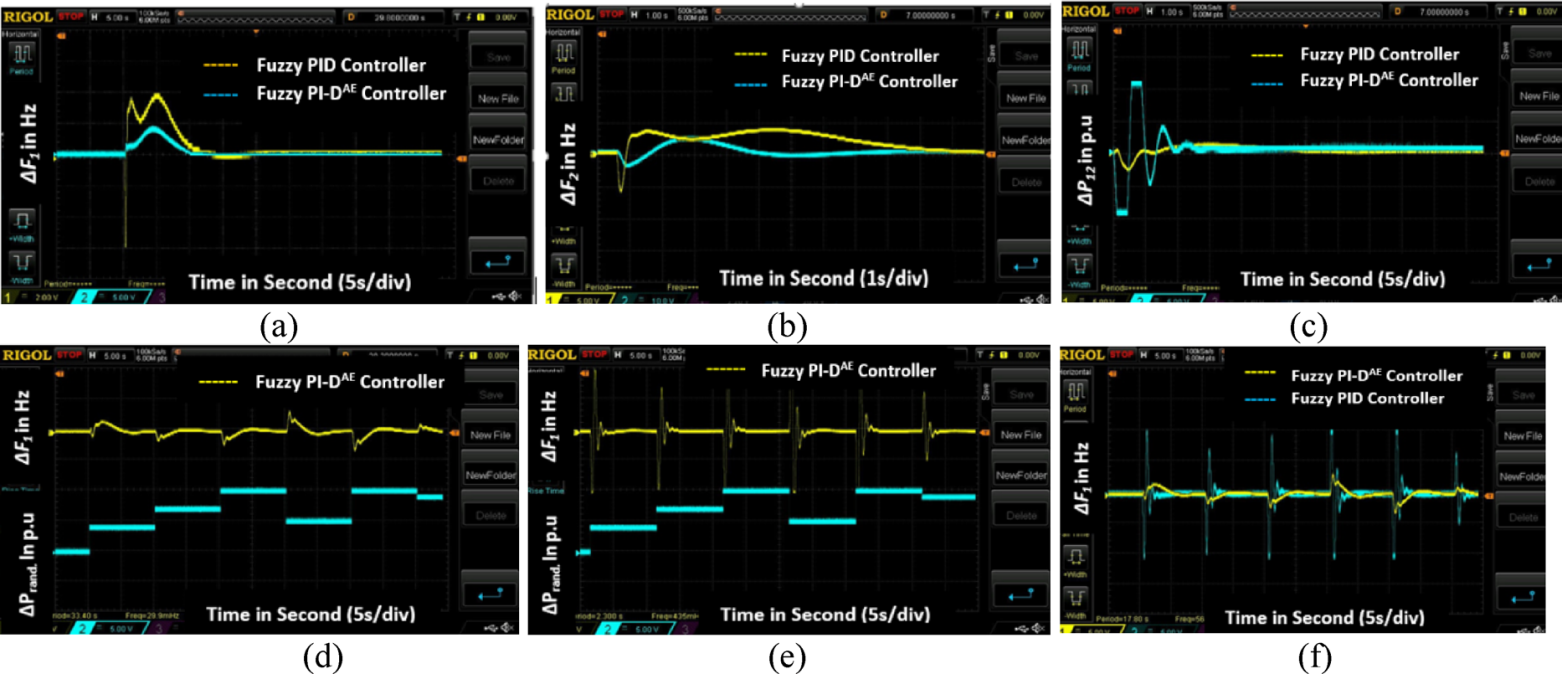
Real-time based responses of (**a**) Area1 frequency deviation under SLP (**b**) Area2 frequency deviation under SLP (**c**) Tie-line power digression under SLP (**d**) Fuzzy PI-D^Æ^ controlled area1 frequency deviation under RLP (**e**) Fuzzy PI-D^Æ^ controlled area1 frequency deviation under RLP (**f**) Comparison of *ΔF*_*1*_ response under RLP.

The complete experimental setup of this research work is illustrated in Fig. 12. The microgrid model and proposed approaches are verified in the real-time platform through T-HIL test bench. The model was run for a real-time interval of 20s with two different load uncertainty situations, such as (i) step load disturbance (ii) random load disturbance. A step load disturbance (SLP) of 1% is injected at area1 and the controlled responses of microgrid frequency and tie-line power are captured in DSO. The Fig. 13a displays the dynamic frequency response of area1 with implementing the proposed Fuzzy PI-D^Æ^ controller and conventional Fuzzy PID controller. In the same sequence, the dynamic responses of area2 frequency and tie-line power are illustrated in Fig. 13b and Fig. 13c respectively. It is clearly observed from the responses that the proposed Fuzzy PI-D^Æ^ outperforms to the conventional Fuzzy PID controller for obtaining frequency stability under a step load disturbance. Further, the dynamic control action and proposed microgrid model are validated under a random load disturbance (RLP) in the real-time based simulation platform. The controlled area1 frequency deviation responses by Fuzzy PI-D^Æ^ and Fuzzy PID controllers under the realization of a RLP are depicted in Fig. 13d and Fig. 13e respectively. The mutual representation of area1 frequency deviation responses due to Fuzzy PI-D^Æ^ and Fuzzy PID controllers is depicted in Fig. 13f. This random load experimental study also validates the efficacy of the proposed Fuzzy PI-D^Æ^ approach in concern to frequency stabilization of the microgrid.

The settling time of the frequency deviation responses and tie-line power response of Fig. 13a-c are assembled in Table 8. It is also clearly observed from these numerical outcomes that the proposed Fuzzy PI-D^Æ^ approach reduces the error faster (least settling time) and able to amend quicker in stable performance of the microgrid under step load disturbance.

**Table 8:** Settling time of responses (Fig. 13a-c)).

Responses	Settling time in second	
	Fuzzy PI-D^Æ^ Controller	Fuzzy PID Controller
*ΔF*_*1*_	7.8246	12.4432
*ΔF*_*2*_	6.8046	10.6684
*ΔP*_*12*_	9.7864	15.4672

#### Stability analysis through bode plots

**Fig. 14:**
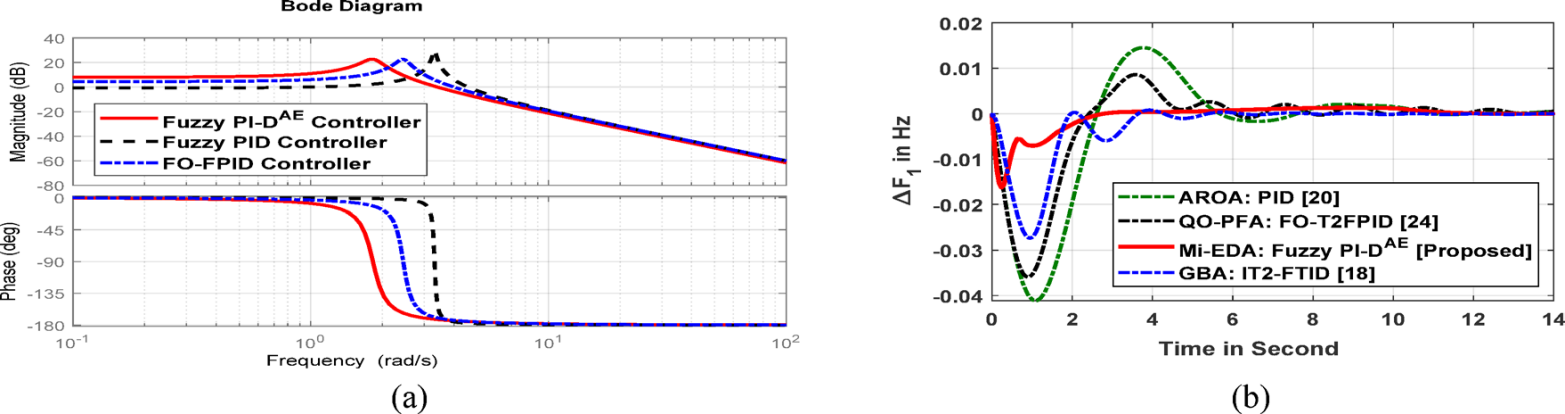
(**a**) Frequency stability using bode plot, (**b**) Dynamic frequency response of microgrid.

The Bode plot assisted frequency domain stability analysis of the Fuzzy PI-D^Æ^ approach is synthesized to verify the effectiveness of the suggested controller. Figure 14 displays the Bode curve of the proposed Fuzzy PI-D^Æ^ controller. Except Fuzzy PI-D^Æ^ approach, the Bode has been traced out with approaching additional control strategies, such as FO-FPID and Fuzzy PID controllers. The Bode plot confirms that, in comparison to other installed controllers, the Fuzzy PI-D^Æ^ controller’s gain margin (GM) and phase margin (PM) are the most positive, crossing both 0^0^ (GM) and − 180^0^ (PM). There is more stability when the GM and PM approach towards a greater positive score.

##### Research findings comparison with recently published research works

This section particularly made a comparison analysis of the outcomes of this research study over a few recently published research findings. Basically, the performance of the recommended Mi-EDA: Fuzzy PI-D^Æ^ approach has been compared with GBA: IT2-FTID [Panda et al., 10], QO-PFA: FO-T2FPID [Bhatta et al., 20] and AROA: PID [Khalil et al.,18] in concern to the microgrid frequency regulation. The regulated frequency response of microgrid by above discussed approaches is illustrated in Fig. 14b. It is clearly conferred from the frequency dynamic response that the anticipated Mi-EDA: Fuzzy PI-D^Æ^ approach is concluded to be more effective to resume faster in nominal grid frequency under a step load disturbance.

#### Sensitive work for robust validation

**Fig. 15:**
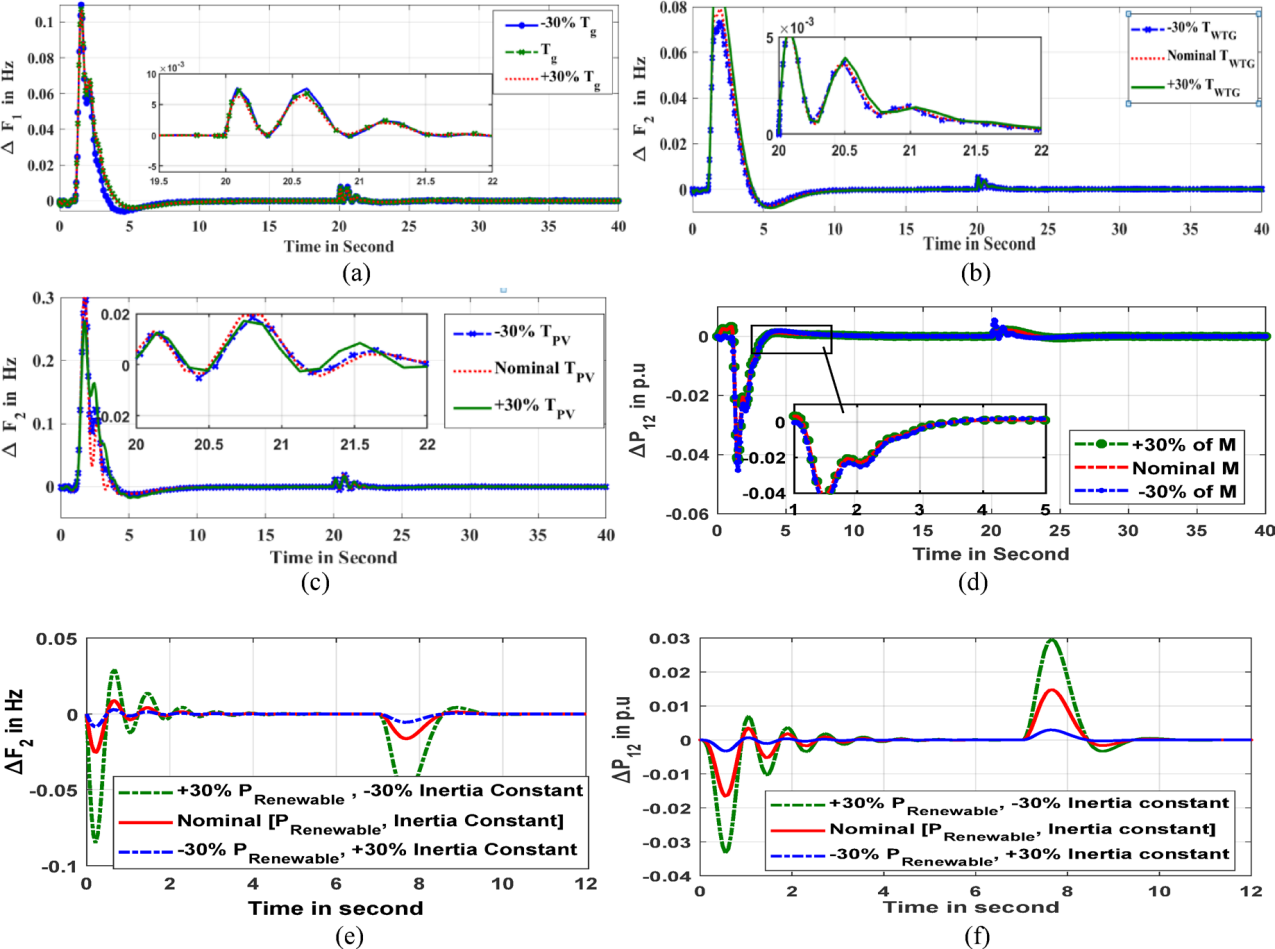
(**a**) Frequency deviancy of area1 (**b**) Frequency deviancy of area2 (**c**) Frequency deviancy of area2 (**d**) Inter-line power oscillation (**e**) Frequency deviancy of area2 under inertia variation (**f**) Inter-line power oscillation under inertia variation.

Resilience is equally as important as excellent controllability when choosing the optimal controller for a certain plant. Typically, various sensitivity analyses are carried out to assess the controller’s resilience. Assessing the system’s performance under controlling system parametric settings while keeping controller parameters constant is the aim of the sensitivity research. The recommended control scheme is deemed to be resilient if, even after a broad range of system parameter adjustments, the system behavior remains unchanged under the specified controller parameters. To test the resilient of the controller, a few model parameters are altered from their original values by ± 30%. These include the thermal plant time constant (*Tg*), wind generator time constant (*T*_*WTG*_), solar PV system time constant (*T*_*PV*_), and microgrid system inertia constant (*M*). The dynamic response of area1 frequency with variation of ‘*T*_*g*_*’* is shown in Fig. 15a. Similarly, the changes in area2 frequency with a noteworthy shift in ‘*T*_*WTG*_*’* and ‘*T*_*PV*_*’*, respectively, are depicted in Figs. 15b,c. The Fig. 15d shows the tie-line power digression reaction to the dip variation of ‘*M*’. The Mi-EDA: Fuzzy PI-D^Æ^ approach is utilized to replicate all of the aforementioned responses within the system. The resulted responses are similar in shape or with little deviation from the nominal parameter based response.

The Table 9 compiles the reaction settling time, suggested ITAE objective function values, maximum overshoot (M.O.) and minimum undershoot (M.U.) of the aforementioned responses. The numerical outcomes addressed in Table 8 are extremely close approximation and validates resilient behaviors of the suggested Fuzzy PI-D^Æ^ controller. It has been demonstrated that the proposed Fuzzy PI-D^Æ^ controller is robust in terms of reducing errors and establishing stability in the hybrid microgrid system.

**Table 9:** Characteristic measures for sensitive results.

Parameter Variation	Percentage Change	Characteristic Parameters			Responses	ITAE
		St (in sec.)	M.O	M.U		
Loading regulation	Nominal	5.82	0.24	-0.18	ΔF_1_	0.1568
	+ 30%	5.84	0.22	-0.16		0.1472
	-30%	5.80	0.24	-0.16		0.1466
*T*_***g***_ regulation	Nominal	6.82	0.12	-0.001	ΔF_1_	0.1522
	+ 30%	6.64	0.11	-0.002		0.1492
	-30%	6.70	0.12	-0.001		0.1518
*T*_***WTG***_ regulation	Nominal	9.68	0.07	-0.142	ΔF_2_	0.1476
	+ 30%	9.76	0.10	-0.150		0.1514
	-30%	9.80	0.07	-0.168		0.1422
*M* regulation	Nominal	16.42	0.12	-0.036	ΔP_12_	0.1502
	+ 30%	16.54	0.13	-0.038		0.1576
	-30%	16.48	0.14	-0.036		0.1524

The sensitivity analysis is also synthesized under a real-world based system variation and an injection of random load disturbance. In a practical system, the increased in renewable energy source based power plants penetration affects the system inertia and reduces abruptly. Owing to such inertia variation concept, this research work addressed the robust analysis with sensitivity regulation of renewable energy-based microgrid power and corresponding inertia constant. In this sensitive study, a simultaneous variation in renewable energy-based electrical power and inertia constant is occurred to testify robustness of the Fuzzy PI-D^Æ^ controller. While the power is enhanced by 30%, the inertia is reduced with 30%. The positive sign (+) is the indication of increased in system parameter, however negative sign (-) stands for reduction in system parameter value. This regulation strategy is accomplished in coordination with practically renewable energy and inertia relationships. The system performances (frequency and tie-line power) are investigated in three different parametric situations such as at nominal state (both renewable energy-based power and inertia constants are kept rated values), increased inertia constant (30% reduction in renewable energy-based power and 30% enhance in inertia constant) and reduced inertia constant (30% enhance in renewable energy-based power and 30% reduction in inertia constant). In such parameter regulating scenarios, the unique Fuzzy PI-D^Æ^ controlled dynamic responses of area2 frequency deviation and change in tie-line power are depicted in Fig. 15e and Fig. 15f respectively. The response with increased inertia exhibits faster stability as compared to the nominal parameter situation. Further, the system response takes time to settle at reduced inertia situation including greater oscillation. This confers resiliency attribute of the prosed Fuzzy PI-D^Æ^ controller under sensitive variation based real world operative situation.

**Fig. 16:**
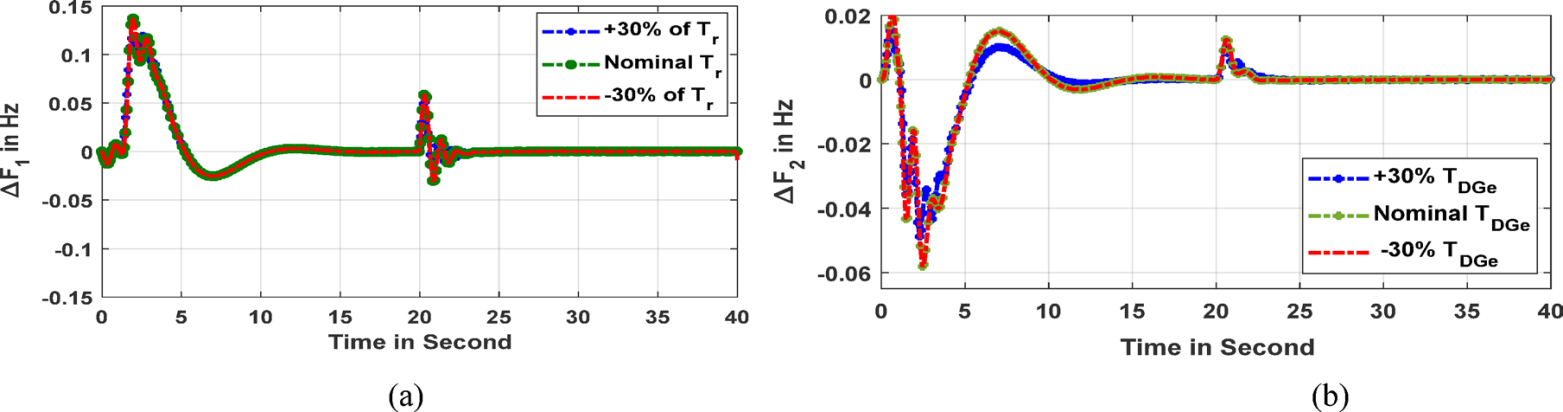
(**a**) Frequency deviancy of area1 (**b**) Frequency deviancy of area2.

Further, the robustness of the recommended Fuzzy PI-D^Æ^ controller has been examined under the incorporation of a time delay response of 0.2 s in the microgrid model. In such a scenario, the dynamic response of the area1 frequency under the variation of the re-heat turbine time constant (*T*_*r*_) by ± 30% from it’s nominal value is depicted in Fig. 16a. Further, a deviation of ± 30% in diesel generator time constant (*T*_*DGe*_) is exercised to develop area2 frequency response and has been illustrated in Fig. 16b. It has been clearly conferred from both frequency responses that variations of ‘*T*_*r*_’ and ‘*T*_*WTG*_*’* have no influence on the frequency response of the microgrid under a unique Fuzzy PI-D^Æ^ controller’s parameter. This validates the robustness of the recommended Fuzzy PI-D^Æ^ controller.

## Conclusion

The performances of the anticipated Fuzzy adaptive exponent PID (Fuzzy PI-D^Æ^) and Math inspired Expansion Distribution algorithm (Mi-EDA) are found to be more effective to obtain frequency stabilities in the microgrid. The recommended Fuzzy PI-D^Æ^controller is able to mitigate the error response faster, according to various responses and numerical findings. Furthermore, compared to standard Fuzzy FO-PID, Fuzzy PID and PID controllers, the proposed Fuzzy PI-D^Æ^ controller can improve the settling time of microgrid’s area 1 frequency deviation (*∆F*_*1*_) by 40.90%, and 109.10% and 168.18% respectively. In terms of methodology, it has been concluded that the proposed Mi-EDA technique exhibits uniqueness and improves the objective function ITAE over the traditional SCA and PSO approaches by 70.74% and 227.18% respectively. The action of chargeable batteries and additional energy-storing equipment promptly advances the frequency stability. Robust analysis shows that the performance of the suggested Fuzzy PI-D^Æ^ controller is unaffected by variations in model parameters, indicating the most resilient behavior of the controller. The system frequency is adversely affected when EVs are linked with microgrids, especially during charging periods. But the proposed Fuzzy PI-D^Æ^ controller can swiftly return to a stable, desired frequency while tactfully handling this disturbance. The EV acts as a backup emergency system in the case of grid integrated discharging and can sustain the output of other generating stations effectively. This novel research work may be extended with considering virtual inertia concepts in the inertia free microgrid environment. Model predictive adaptive controllers and improved optimization algorithms may be proposed to improve stability behavior of the islanded microgrid. The stability study of the proposed controller may be synthesized by developing state-space model of the proposed microgrid system.

## Electronic supplementary material

Below is the link to the electronic supplementary material.

Supplementary Material 1

## Data Availability

The necessary data of this research work will be available on request. The corresponding authors will be contacted for providing data for this study.

## Abbreviations

*EDA*: Exponential distribution algorithm
*AC*: Alternating current
*ACE*: Area control error
*AGC*: Automatic generation control
*DEG*: Diesel engine generator
*DG*: Distributed generation
*EV*: Electric vehicle
*FACTS*: Flexible AC transmission system
*PI-D*^Æ^: Adaptive exponential PID
*GA*: Genetic algorithm
*MATLAB*: Matrix laboratory
*MW*: Megawatt
*NB*: Negative big
*NS*: Negative small
*PSO*: Particle swarm optimizer
*PID*: Proportional integral derivative
*PB*: Positive big
*PS*: Positive small
*RES*: Renewable energy sources
*V2G*: Vehicle to grid
*Z*: Zero
*SOC*: State of charge

## Parameters

*K*_*WTG*_: Wind generator gain
*K*_*FC*_: Fuel cell gain
*K*_*AE*_: Aqua electrolyzer gain
*K*_*WTG*_: Wind generator gain
*K*_*FC*_: Fuel cell gain
*K*_*AE*_: Aqua electrolyzer gain
*K*_*MiT*_: Micro turbine gain
*K*_*T*_: PV system gain
*K*_*DGe*_: Diesel generator gain
*K*_*BEnS*_: Battery system gain
*K*_*UC*_: Ultra capacitor gain
*T*_*WTG*_: Time constant (Tc) of wind-gen
*T*_*AE*_: Tc of aqua electrolyzer
*T*_*DG*_: Tc of diesel generator
*LFC*: Load frequency Control
*T*_*MiT*_: Tc of micro turbine
*T*_*T*_: Tc of PV system
*T*_*EV*_: Tc of electric vehicle
*T*_*BEnS*_: Tc of battery system
*T*_*FEnS*_: Tc of flywheel energy
*T*_*U*_: Tc of ultra-capacitor
*T*_*GeT*_: Tc of geo-thermal
*M*: System angular momentum
*D*: System damping coefficient
*ΔF*: Frequency deviation
*K*_*H*_: Hydro system strength factor
Æ: Adaptive exponential
