# Supplementary material for: Resilient math inspired EDA optimized fuzzy adaptive exponent controller for LFC improvement of an EV integrated microgrid
Source: Sci Rep. 2025 Aug 5;15:28635. doi: 10.1038/s41598-025-12275-1 (PMC12325654; doi:10.1038/s41598-025-12275-1)
Supplement: Supplementary file 1 — Supplementary Material 1 [file 41598_2025_12275_MOESM1_ESM.docx]

**Appendix-A**

*Thermal station :* Tc = Time constant; T_g_ (Tc of governor)= 0.08s; T_r_ (Tc of Turbine) =10s ; Kr (Gain of reheat turbine) =0.2 ; T_t_ (Tc of Non-reheat turbine)= 0.3s; GRC (Generation rate constraint) = ±3%/min. ; K_T_ = Strength factor = 0.45.

*Hydro station :* T_rh_ (Tc of governor) = 0.2s; T_gh_ (Tc of Turbine) =5s ; T_W_ (water recovery time) =1s; GRC (Generation rate constraint) = 270%/min. ; K_H_ = Strength factor = 0.35*. Gas station parameters:* b_g_ = 0.5; c_g_ = 1; X_C_ = 0; Y_C_ = 1; T_cr_ = 0.03; T_f_ = 0.23s; T_cd_ = 0.2s; K_G_ =0.1. *Nuclear Energy Centre:* T_gN_ (Tc of governor)=1.4s ; T_t_ (Tc of Turbine) = 1s; Kh (Turbine’s gain = 1. *Microgrid Details:* T_WTG_ (Tc of wind generator) = 1.5 s; T_PV_ (Tc of PV set) = 1.8 s; T_DEG_ (Tc of diesel generator) = 2 s; T_MT_ (Tc of micro-turbine setup) = 2 s;T_G_ (Tc of geo-thermal generator) = 1.2s; T_FC_ (Tc of fuel-cell system) = 0.2s; D (Damping coefficient) = 0.012; M( Constant of inertia) = 2.

**Appendix-B**

*Data of total energy model (TEM) & EV System:*

*T_EV_* = Electric vehicle time constant= 1s; $E_{control}^{min}$ = Energy of all EV = 60 kwh ; $C_{Kwh}^{*}$ = 15kwh; *N_plug-out_* = Driving EV numbers =2; *N_control_* = Controllable electric vehicle numbers = 4 ; *N_initial_* = Number of initial EVs =6.
